# Supplementary material for: Redefining the Limits of Functional Continuity in the Early Evolution of P-Loop NTPases
Source: Mol Biol Evol. 2025 Mar 12;42(4):msaf055. doi: 10.1093/molbev/msaf055 (PMC11959459; doi:10.1093/molbev/msaf055)
Supplement: msaf055_Supplementary_Data [file msaf055_supplementary_data.pdf]

Supplementary Materials for:

Redefining the Limits of Functional Continuity in the Early Evolution of P-Loop NTPases

Andrey O. Demkiv[1], Saacnicteh Toledo-Patiño,[2] Encarnación Medina-Carmona,[3] Andrej Berg,[1] Gaspar P. Pinto,[1] Antonietta Parracino,[1] Jose M. Sanchez-Ruiz,[3] Alvan C. Hengge,[4] Paola Laurino,[2,5,] Liam M. Longo,[6,7,‡] and Shina Caroline Lynn Kamerlin[8,9‡]

1. Department of Chemistry – BMC, Uppsala University, BMC Box 576, S-751 23 Uppsala, Sweden.

2. Protein Engineering and Evolution Unit, Okinawa Institute of Science and Technology, Graduate University (OIST), Okinawa, 904-0495, Japan.

3. Departamento de Química Física, Facultad de Ciencias, Unidad de Excelencia de Química aplicada a Biomedicina y Medioambiente (UEQ), Universidad de Granada, 18071 Granada, Spain.

4. Department of Chemistry and Biochemistry, Utah State University, Logan, Utah 84322-0300, United States.

5. Institute for Protein Research, Osaka University, Suita, Japan.

6. Blue Marble Space Institute of Science, Seattle, Washington 98104, United States.

7. Earth-Life Science Institute, Institute of Science Tokyo, Tokyo, 152-8550, Japan.

8. School of Chemistry and Biochemistry, Georgia Institute of Technology, 901 Atlantic Drive NW, Atlanta, GA 30332, USA.

9. Department of Chemistry, Lund University, Box 124, 221 00 Lund, Sweden.

Corresponding Authors' Email Addresses: llongo@elsi.jp, skamerlin3@gatech.edu

Supplementary Methods

System Preparation and Equilibration Procedure for Hamiltonian Replica Exchange Molecular Dynamics Simulations

All systems were placed in either triclinic solvent boxes of TIP3P (Jorgensen, et al. 1983) water molecules, or methanol (Caldwell and Kollmann 1995), with box lengths defined so that all atoms were at least 10 Å from the box edges. All simulations were performed using periodic boundary conditions, with long-range electrostatics being handled using the Particle Mesh Ewald (PME) approach (Darden, et al. 1993), and a 10 Å cutoff. K^+ and Cl^- counterions were added to each system to neutralize the charge of each peptide/complex, and to give a final ion concentration of 0.1 M. All simulations were performed using a 2 fs time step, with all bonds containing hydrogen atoms being constrained using the SHAKE algorithm (Ryckaert, et al. 1977).

Each system was first minimized using 500 steps of unrestrained steepest descent minimization, followed by 5000 steps of conjugate gradient minimization with constraints on all bonds with hydrogen atoms. At this point, the system was then heated from 0 to 300 K over 400 ps of simulation time in an NVT ensemble, with temperature and pressure regulated using velocity rescaling (Bussi, et al. 2007) (collision frequency of 1 ps). Following this, a further 400 ps of equilibration was performed at 300 K in an NPT ensemble, once again using velocity rescaling and the Parrinello-Rahman barostat (Parrinello and Rahman 1980), with 1000 kJ mol^{-1} nm^{-1} harmonic restraints applied to all heavy atoms of the peptide. Both NVT and NPT equilibration simulations were performed using a time step of 2 fs and the P-LINCS (Hess 2008) algorithm for restraining all bonds to hydrogen atoms.

Finally, in the case of the end-restrained HREX-MD simulations, an additional 4184 kJ mol^{-1} nm^{-1} harmonic restraint was put on the $C_{\alpha 1}$ - $C_{\alpha 8}$ distance corresponding to the distance of the respective situated structure (see **Supplementary Table 17**).

Supplementary Figures

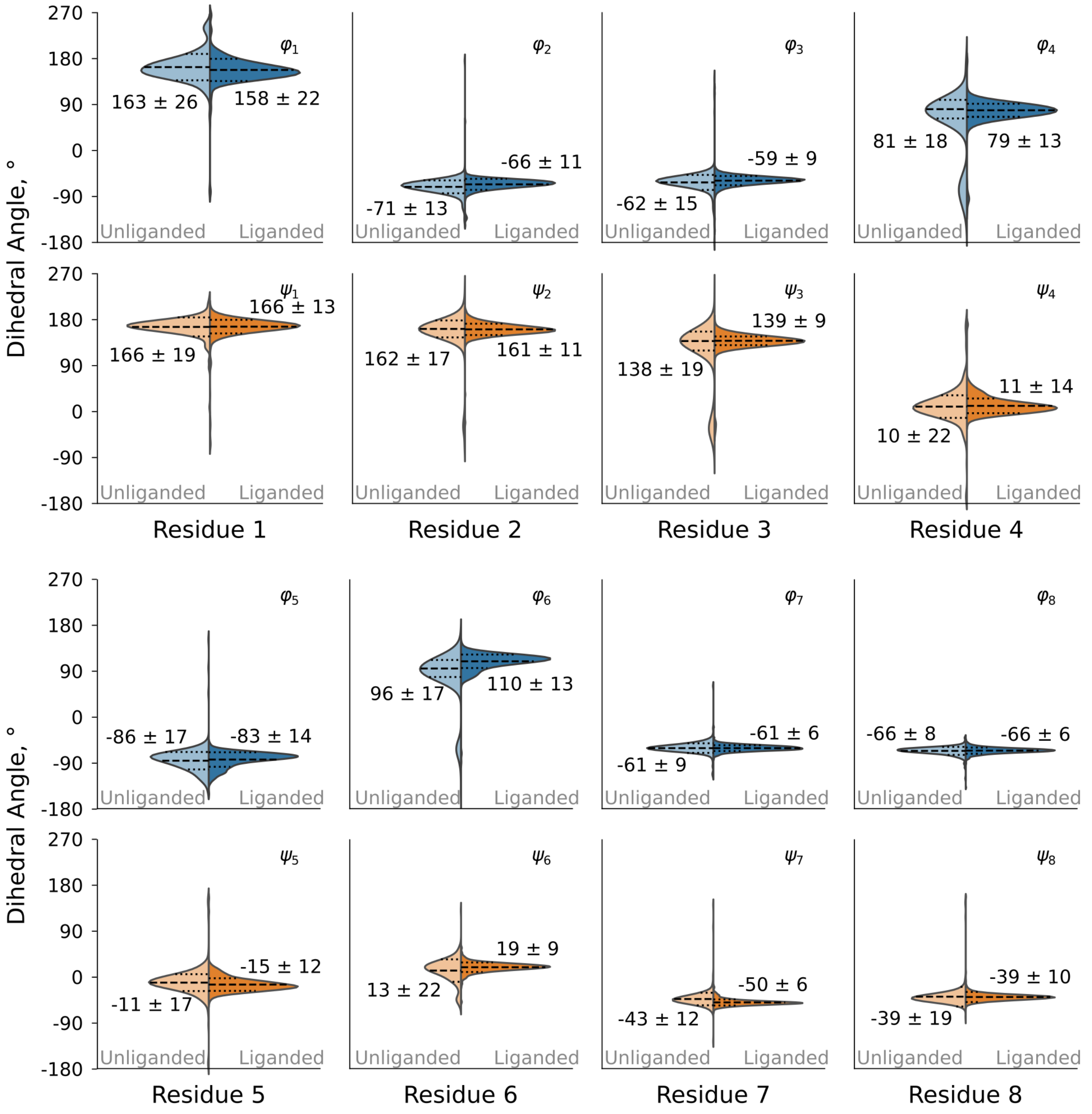

Supplementary Figure 1: Analysis of Walker A P-loop conformational change upon phospho-ligand binding. Shown here are the distributions of the φ- and ψ-dihedral angles for representative liganded or unliganded domains. Dashed and dotted lines indicate the mean value ± 1σ.

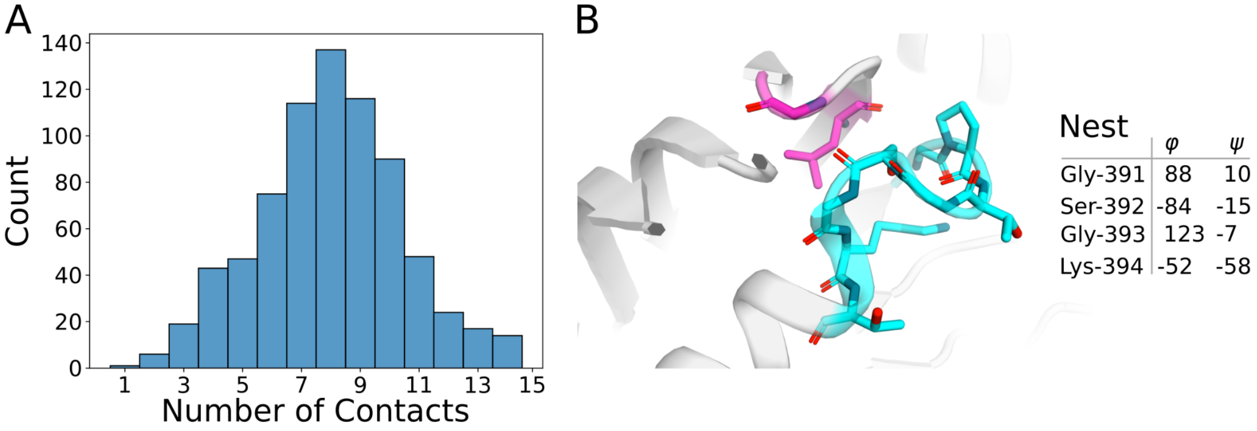

Supplementary Figure 2: Supporting interaction analysis. A. Distribution of the number of heavy atom contacts formed by situated Walker A motifs with the surrounding protein structure. *α*-helical contacts, which are formed by the C-terminal residues of the situated Walker A motif, and interactions to covalently connected adjacent residues, were excluded from the analysis as they are largely invariant. The cutoff distance used for calculating contacts was 3.5 Å. **B**. Example of a P-loop with a canonical conformation and a small number of supporting contacts (ECOD domain e4q7mB1, with two supporting contacts). The P-loop is shown in cyan and residues within 3.5 Å that are not related to propagation of the *α*-helix are shown in magenta. Backbone *φ*- and *ψ*-dihedral angles of the nest-forming residues in this structure are shown in the inset table.

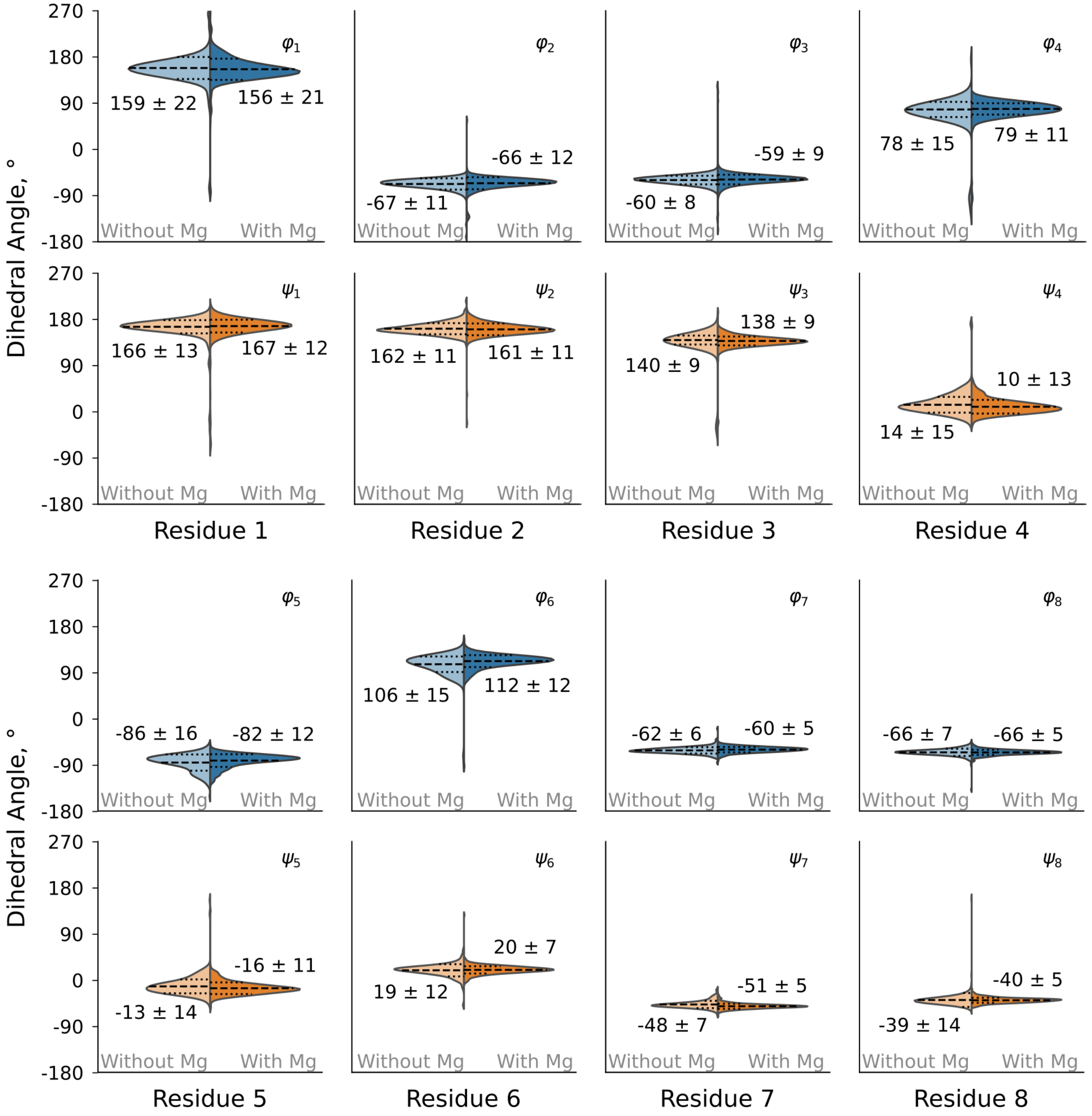

Supplementary Figure 3: Analysis of Walker A P-loop conformational change upon Mg^{2+} binding. Shown here are the distributions of the *φ*- and *ψ*-dihedral angles for representative structures with and without a bound Mg^{2+}. Dashed and dotted lines indicate the mean value ± 1σ.

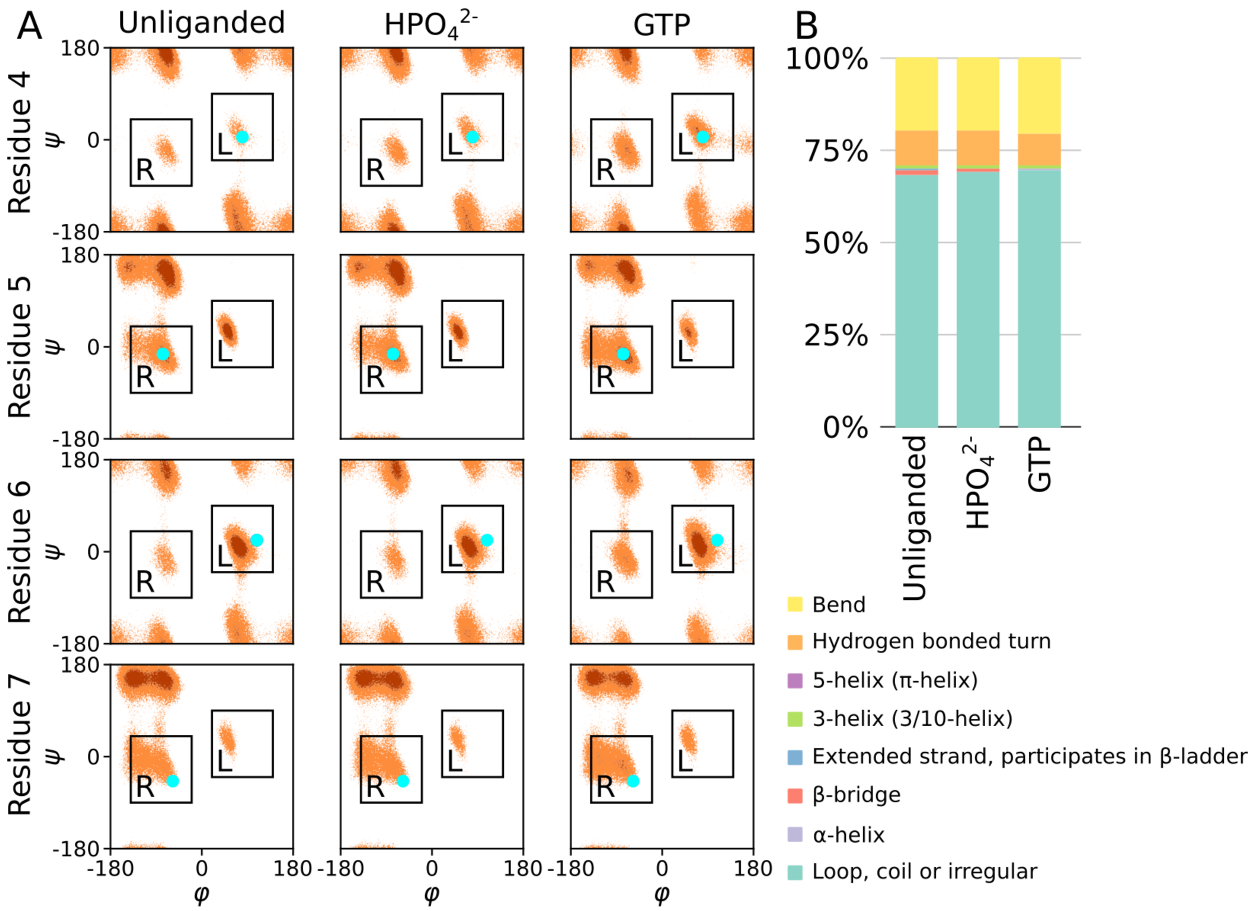

Supplementary Figure 4: Conformational dynamics of the Walker A-derived hexapeptide SGAGKT. Data based on 1 µs HREX-MD simulations in the presence (HPO_4^{2-} or GTP) or absence of a ligand. **A.** Calculated Ramachandran plots. The values for the situated structures are indicated with a cyan dot. **B.** Secondary structure composition calculated using MDTraj (McGibbon, et al. 2015) and presented using DSSP (Kabsch and Sander 1983) annotations.

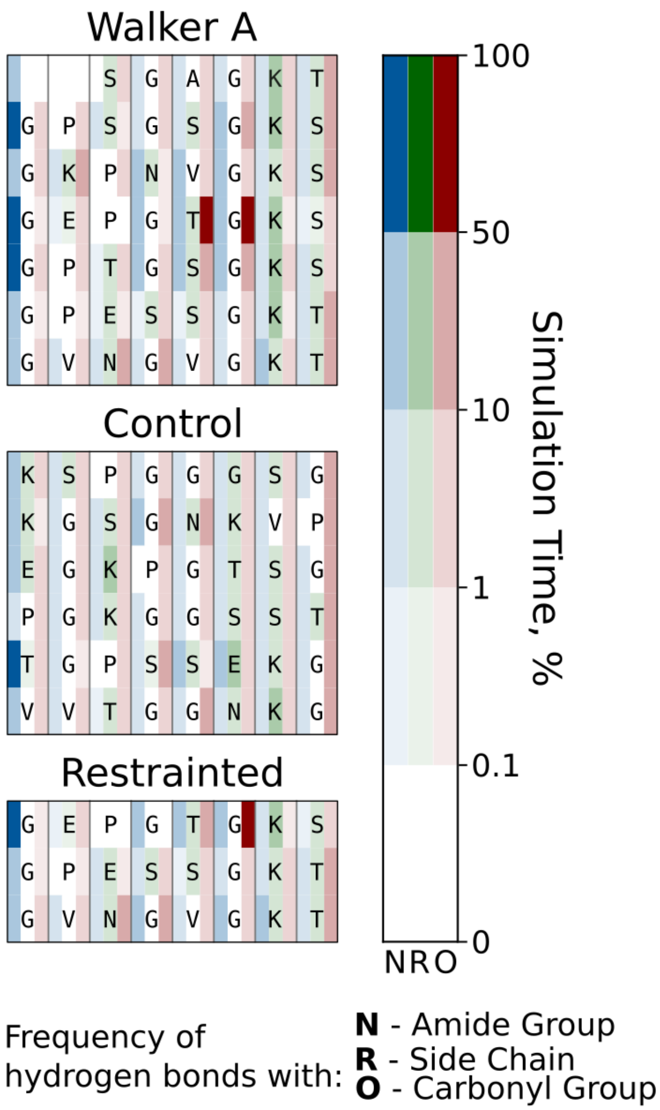

Supplementary Figure 5: **Interaction profile of the Walker A peptides with the base and sugar groups of GTP**. Hydrogen bond donor-acceptor distance and angle cutoffs were 3.5 Å and 135°, respectively. The raw data for this figure are shown in **Supplementary Tables 3, 8** and **11.**

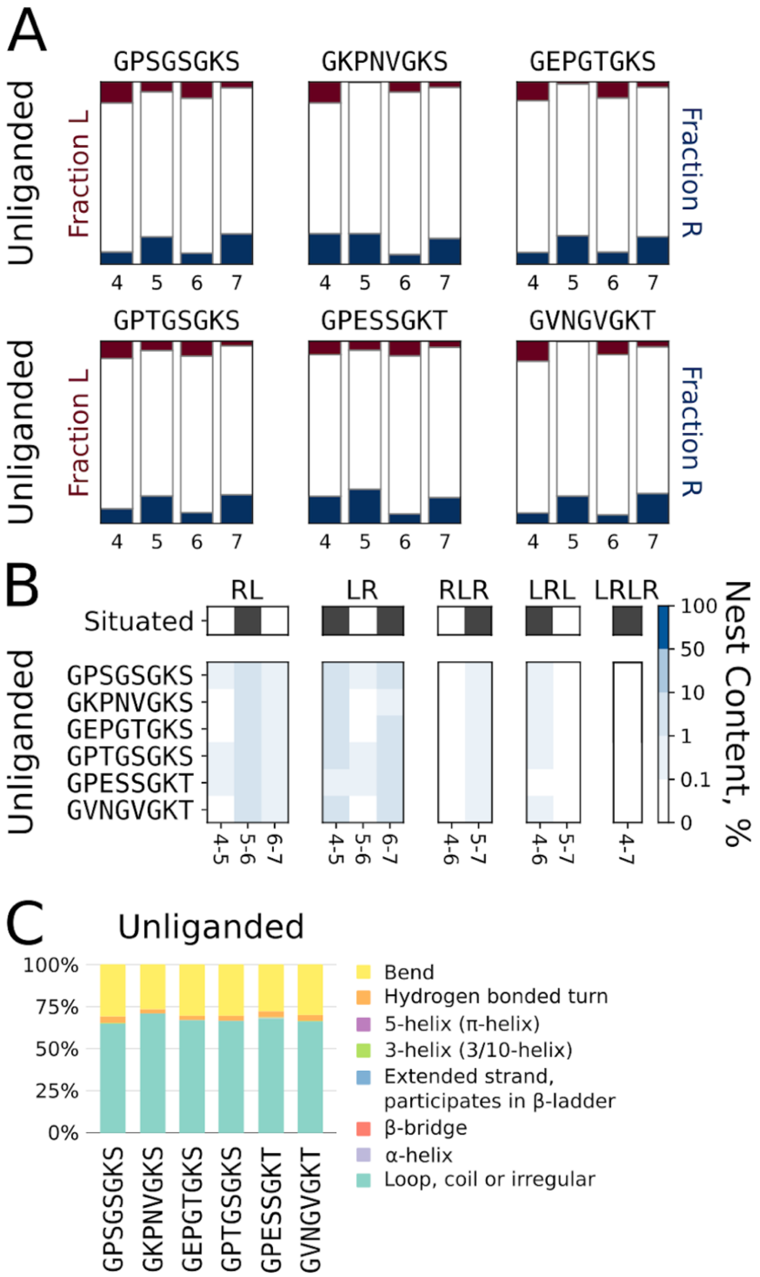

Supplementary Figure 6: Conformational dynamics of the Walker A-derived octapeptides during simulations using the CHARMM36m force field (Huang, et al. 2016). **A.** Uncorrelated preferences for α_L and α_R backbone dihedrals. **B**. Occurrence of correlated stretches of α_L and α_R conformations. **C**. Secondary structure calculated by MDTraj (McGibbon, et al. 2015) and presented using DSSP (Kabsch and Sander 1983) annotations. The raw data for panel B are shown in **Supplementary Table 6.**

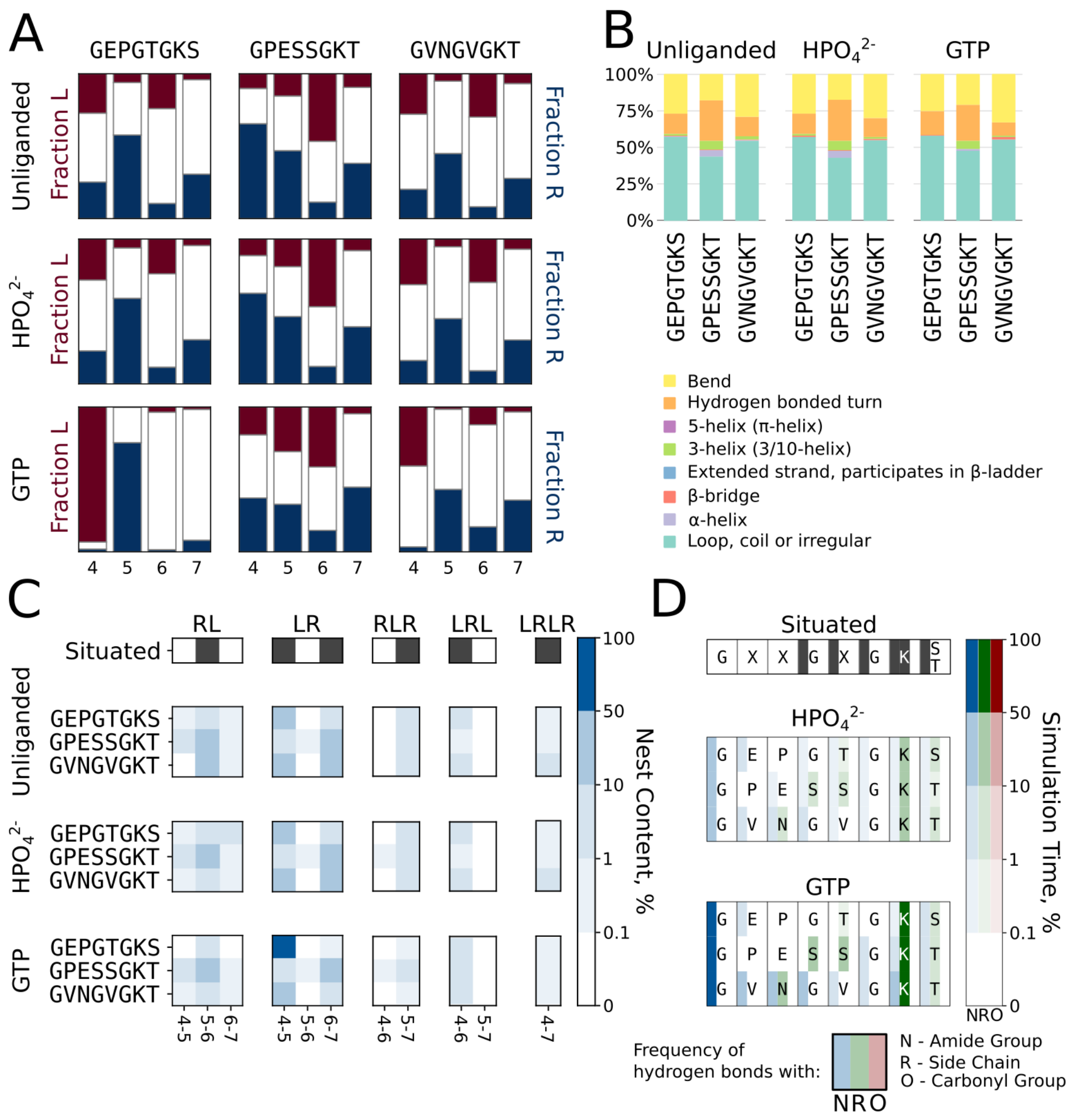

Supplementary Figure 7: Conformational dynamics of the Walker A-derived octapeptides in simulations in which the C$_{\alpha 1}$-C$_{\alpha 8}$ distance of the peptide is restrained. A. Uncorrelated preferences for α_L and α_R backbone dihedral angles. Secondary structure calculated by MDTraj (McGibbon, et al. 2015) and presented using DSSP (Kabsch and Sander 1983) annotations. **C**. Occurrence of correlated stretches of α_L and α_R conformations. **D**. Interaction profile of the peptides with a ligand. Hydrogen bond donor-acceptor distance and angle cutoffs were 3.5 Å and 135°, respectively. The raw data for panels C and D are shown in **Supplementary Tables 7** and **8.**

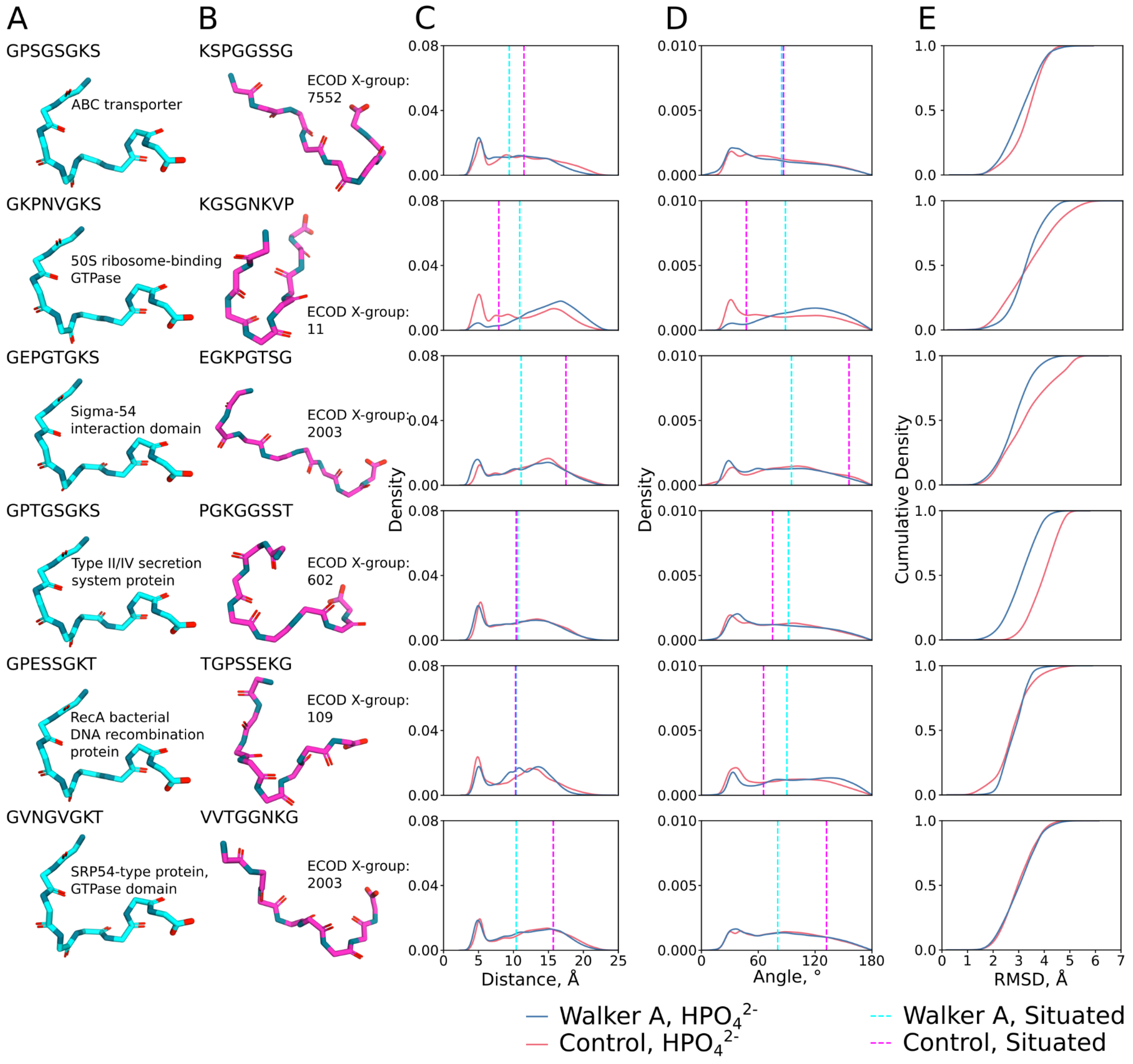

Supplementary Figure 8: Conformational dynamics of the control octapeptides in presence of HPO_4^{2-}. A. Situated structures of the Walker A-derived octapeptides. **B**. Situated structures of control octapeptides. **C**. Distribution of $C_{\alpha 1}$-$C_{\alpha 8}$ distances for the free peptide in the presence of a phosphate ligand. **D**. Distribution of $C_{\alpha 1}$-N_5-$C_{\alpha 8}$ angles for the free peptide in the presence of a phosphate ligand. The situated conformation for panels C and D is indicated with a dotted line. **E**. Cumulative distribution of the root mean square deviations (RMSD, Å) of each set of simulations in the presence of orthophosphate relative to the situated structure.

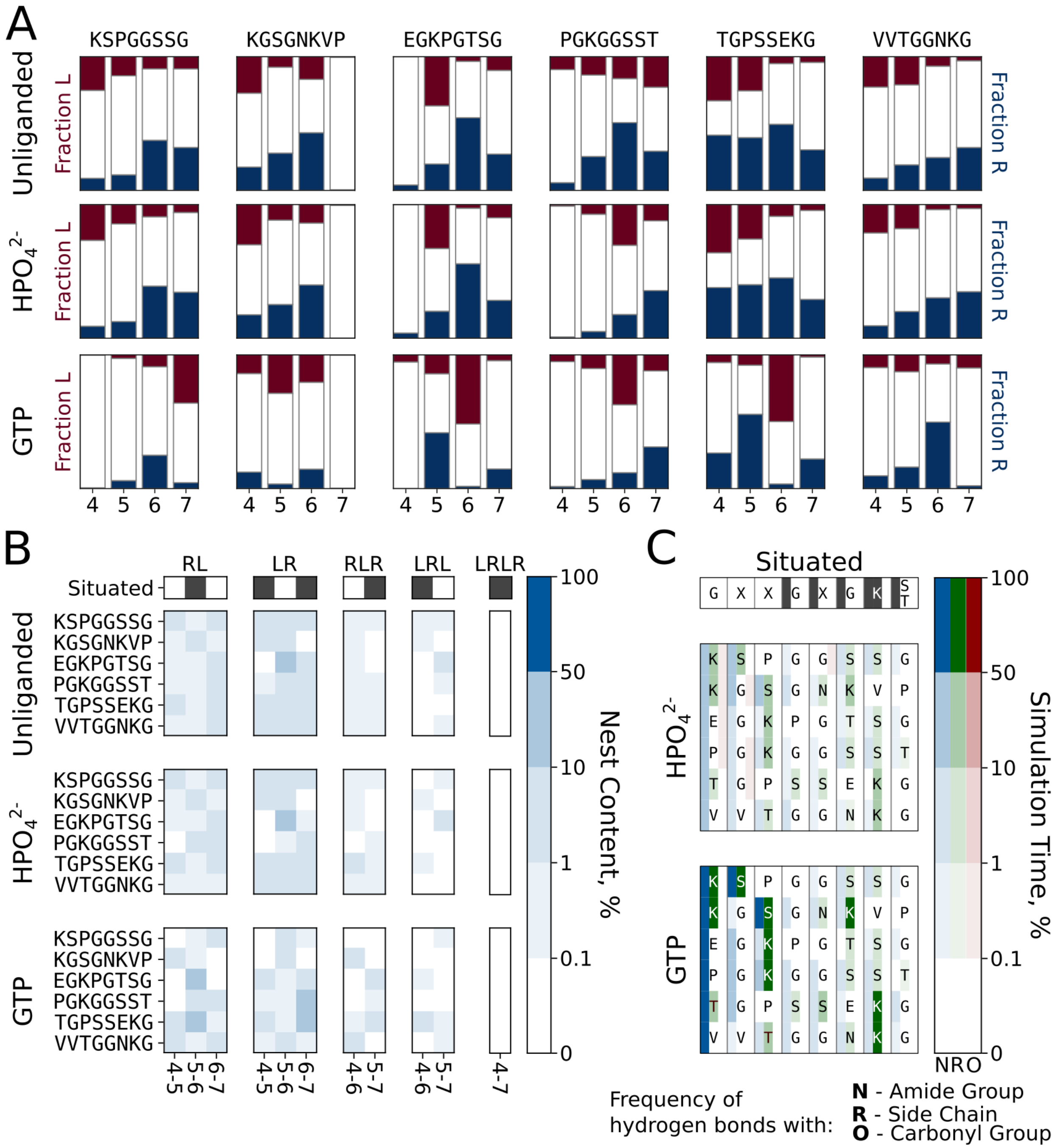

Supplementary Figure 9: **Conformational dynamics of control octapeptides. A**. Uncorrelated preference for α_L and α_R backbone dihedrals. **B**. Occurrence of correlated stretches of α_L and α_R conformations. **C**. Interaction profile of the peptides with a ligand. The raw data for panels B and C are shown in **Supplementary Tables 10** and **11.**

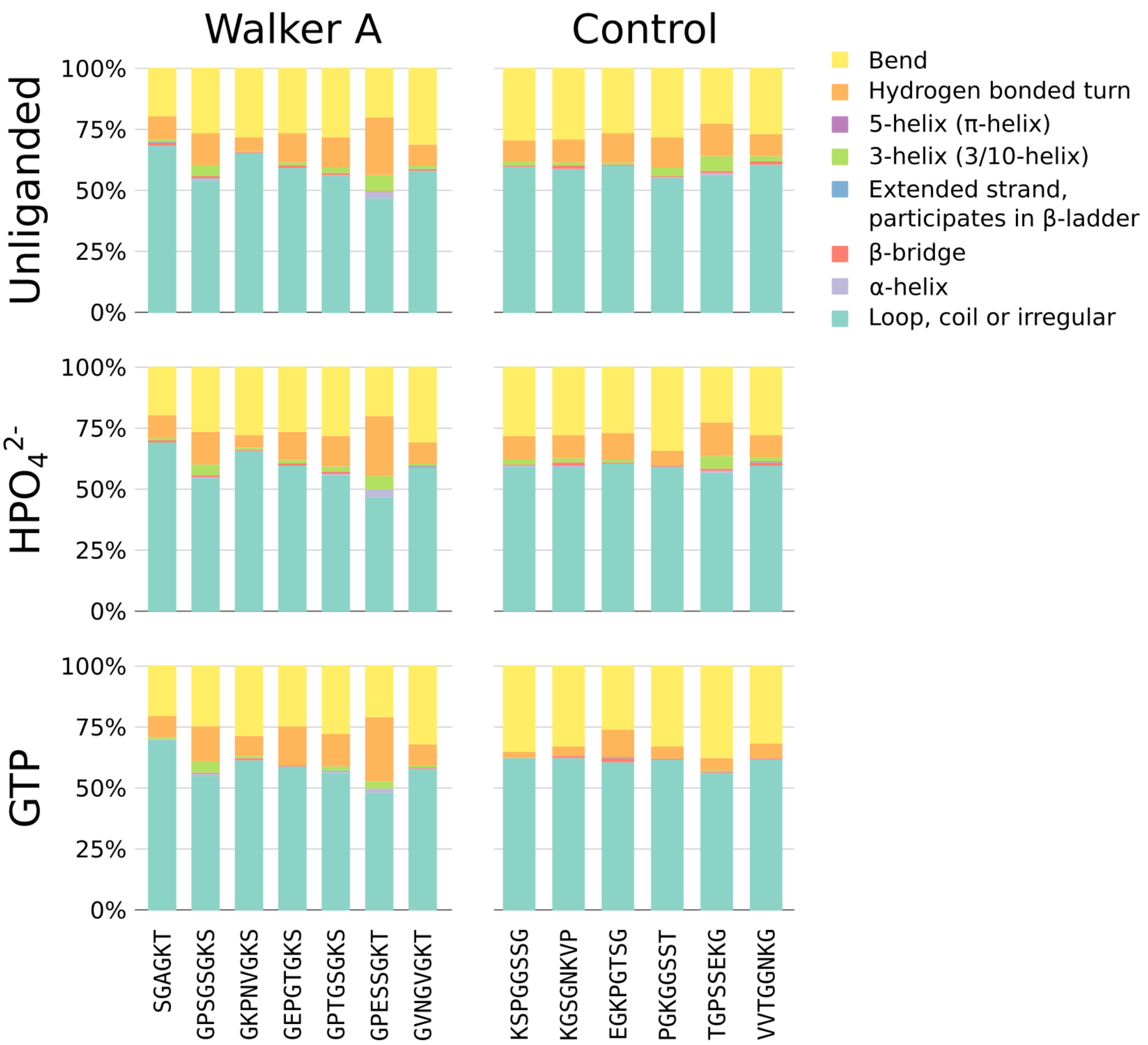

Supplementary Figure 10: **Secondary structure compositions for the disembodied Walker A peptides and the control peptides in the presence (HPO_4^{2-} or GTP) or absence of a ligand**. Calculated using MDTraj (McGibbon, et al. 2015) and presented using DSSP (Kabsch and Sander 1983) annotations.

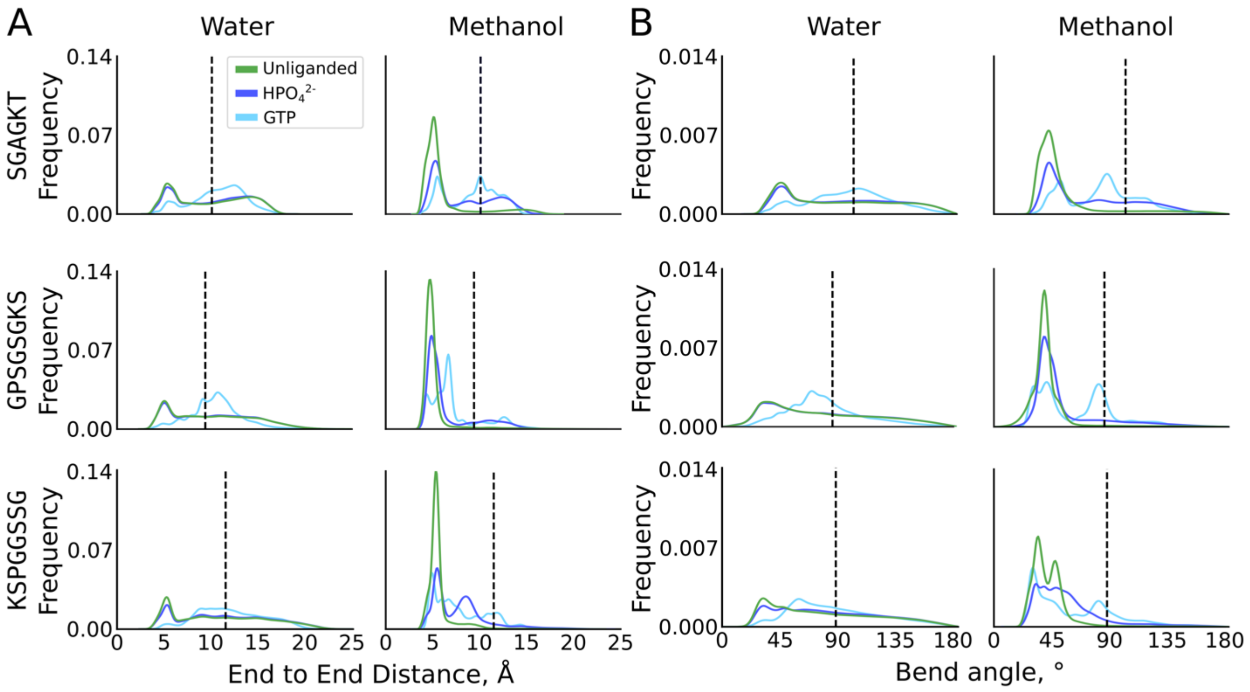

Supplementary Figure 11: The impact of solvent on peptide structure. Comparison of the distribution of the **A**. $C_{\alpha1}$-$C_{\alpha6}$ distances and **B.** $C_{\alpha1}$-N_4-$C_{\alpha6}$ angles for the SGAGKT hexapeptide, and the GPSGSGKS (Walker A) and KSPGGSSG (shuffle control) octapeptides, simulated in water and methanol.

Supplementary Tables

Supplementary Table 1: Residues that interact with the Mg^{2+} ion in the representative P-loop structures.

**Residue**	G	X	X	G	X	G	K	S/T
**Count**	1	0	5	2	1	1	4	419

Supplementary Table 2: Occurrence (% simulation time) of correlated stretches of α_L and α_R conformations in Walker A-derived octapeptides

Sequence	4-5 (RL)	5-6 (RL)	6-7 (RL)	4-5 (LR)	5-6 (LR)	6-7 (LR)	4-6 (RLR)	5-7 (RLR)	4-6 (LRL)	5-7 (LRL)	4-7 (LRLR)
**Unliganded**											
**SGAGKT**	0.22	2.63	0.55	1.03	0.13	5.33	0.02	0.72	0.09	0.02	0.02
**GPSGSGKS**	0.80	7.76	0.61	4.57	0.65	6.77	0.01	1.68	1.23	0.00	0.22
**GKPNVGKS**	1.72	5.41	0.45	7.07	0.06	3.74	0.01	0.63	1.72	0.00	0.25
**GEPGTGKS**	0.23	17.59	0.63	16.60	0.18	7.49	0.04	2.80	7.10	0.06	1.16
**GPTGSGKS**	0.81	8.66	0.68	4.57	0.68	7.20	0.02	1.83	1.15	0.09	0.23
**GPESSGKT**	9.91	11.08	0.96	1.30	0.46	11.57	0.13	2.25	0.48	0.17	0.19
**GVNGVGKT**	0.39	6.69	0.47	9.58	0.05	6.04	0.00	1.24	1.50	0.03	0.30
**HPO_4^{2-}**											
**SGAGKT**	0.40	2.48	0.50	1.72	0.19	6.32	0.00	0.70	0.19	0.03	0.04
**GPSGSGKS**	0.94	7.03	0.67	4.79	0.64	7.14	0.06	1.76	1.14	0.10	0.22
**GKPNVGKS**	1.51	5.33	0.45	6.03	0.13	5.07	0.07	1.21	1.64	0.00	0.60
**GEPGTGKS**	0.26	15.55	0.70	16.11	0.18	7.41	0.01	2.92	6.85	0.00	1.27
**GPTGSGKS**	0.56	7.42	0.71	4.80	0.80	7.77	0.00	1.79	1.09	0.04	0.23
**GPESSGKT**	8.15	14.43	0.92	3.66	0.54	13.08	0.03	3.33	1.52	0.08	0.40
**GVNGVGKT**	0.59	6.00	0.95	10.17	0.05	6.28	0.00	1.28	1.79	0.00	0.49
**GTP**											
**SGAGKT**	0.49	3.54	0.78	5.53	0.41	13.57	0.02	2.28	1.32	0.00	0.98
**GPSGSGKS**	0.49	3.54	0.78	5.53	0.41	13.57	0.02	2.28	1.32	0.00	0.98
**GKPNVGKS**	0.41	6.12	0.31	20.49	1.05	7.46	0.24	3.49	1.72	0.14	0.91
**GEPGTGKS**	2.36	3.28	0.53	11.79	0.24	4.06	0.00	0.66	0.64	0.00	0.29
**GPTGSGKS**	0.34	1.51	0.06	33.53	2.19	0.96	0.34	0.32	0.98	0.00	0.21
**GPESSGKT**	0.22	4.26	0.15	26.42	0.13	4.37	0.00	0.91	1.02	0.01	0.09
**GVNGVGKT**	6.16	19.97	0.33	3.18	1.89	6.02	1.62	1.42	0.64	0.04	0.09

Supplementary Table 3: Peptide-ligand hydrogen bonding (% simulation time) between the Walker A P-loop sequences studied in this work and either HPO_4^{2-} or GTP.[a]

Sequence		HPO$_4^{2-}$							GTP-α						
		SGAGKT	GPSGSGKS	GKPNVGKS	GEPGTGKS	GPTGSGKS	GPESSGKT	GVNGVGKT	SGAGKT	GPSGSGKS	GKPNVGKS	GEPGTGKS	GPTGSGKS	GPESSGKT	GVNGVGKT
	**N**	12.81	12.78	12.95	9.36	11.54	10.51	12.33	35.50	46.21	41.21	35.03	40.08	29.71	42.76
**1**	**R**		0.00	0.00	0.00	0.00	0.00	0.00		0.00	0.00	0.00	0.00	0.00	0.00
	**O**		0.12	0.13	0.08	0.09	0.05	0.09		0.00	0.00	0.00	0.00	0.00	0.00
	**N**		0.00	3.54	0.97	0.00	0.00	3.19		0.00	19.61	0.62	0.00	0.00	14.86
**2**	**R**		0.00	17.15	0.02	0.00	0.00	0.00		0.00	25.05	0.00	0.00	0.00	0.00
	**O**		0.03	0.17	0.09	0.03	0.01	0.06		0.00	0.00	0.00	0.00	0.00	0.00
	**N**		1.52	0.00	0.00	0.82	0.19	3.50		3.27	0.00	0.00	0.38	0.04	16.81
**3**	**R**	7.91	3.35	0.00	0.00	1.56	0.02	4.40	8.17	4.72	0.00	0.00	0.52	0.00	13.71
	**O**	0.10	0.02	0.00	0.00	0.01	0.00	0.08	0.00	0.00	0.00	0.00	0.00	0.00	0.00
	**N**	7.54	0.78	0.19	0.15	0.55	0.22	1.66	19.71	2.07	0.01	0.00	0.21	0.02	10.36
**4**	**R**	0.00	0.00	0.96	0.00	0.00	5.20	0.00	0.00	0.00	1.53	0.00	0.00	12.15	0.00
	**O**	0.09	0.01	0.00	0.00	0.02	0.01	0.03	0.00	0.00	0.00	0.00	0.00	0.00	0.00
	**N**	1.28	1.32	0.44	0.52	1.58	0.51	0.33	4.47	4.59	0.08	0.00	3.06	0.08	1.87
**5**	**R**	0.00	4.54	0.00	0.74	4.36	4.41	0.00	0.00	7.84	0.00	0.12	8.36	17.45	0.00
	**O**	0.03	0.01	0.01	0.01	0.02	0.01	0.01	0.00	0.00	0.00	0.00	0.00	0.00	0.00
	**N**	0.61	0.80	0.36	0.22	1.06	0.36	0.36	4.33	8.38	0.00	0.32	5.60	0.55	2.36
**6**	**R**	0.00	0.00	0.00	0.00	0.00	0.00	0.00	0.00	0.00	0.00	0.00	0.00	0.00	0.00
	**O**	0.03	0.03	0.01	0.01	0.03	0.02	0.01	0.00	0.00	0.00	0.00	0.00	0.00	0.00
	**N**	0.61	0.72	0.50	0.36	0.70	0.37	0.52	0.28	9.85	4.23	0.43	2.75	1.74	2.55
**7**	**R**	17.88	17.32	11.49	14.46	15.22	14.13	14.01	37.28	44.46	33.81	59.42	48.66	47.39	42.45
	**O**	0.02	0.02	0.01	0.01	0.02	0.01	0.01	0.00	0.00	0.00	0.00	0.00	0.00	0.00
	**N**	0.98	1.19	0.69	0.53	0.99	0.32	0.53	0.46	5.82	2.99	0.59	1.04	1.74	0.64
**8**	**R**	1.33	3.73	1.98	1.99	2.91	0.48	0.90	0.67	8.40	4.07	0.86	1.62	2.17	1.32
	**O**	0.01	0.01	0.01	0.01	0.01	0.01	0.01	0.00	0.00	0.00	0.00	0.00	0.00	0.00

Sequence		GTP-β							GTP-γ						
		SGAGKT	GPSGSGKS	GKPNVGKS	GEPGTGKS	GPTGSGKS	GPESSGKT	GVNGVGKT	SGAGKT	GPSGSGKS	GKPNVGKS	GEPGTGKS	GPTGSGKS	GPESSGKT	GVNGVGKT
	N	22.72	31.42	26.07	46.45	39.77	26.57	30.86	36.46	43.07	41.97	44.31	46.37	43.95	53.56
1	R		0.00	0.00	0.00	0.00	0.00	0.00		0.00	0.00	0.00	0.00	0.00	0.00
	O		0.00	0.00	0.00	0.00	0.00	0.00		0.00	0.00	0.00	0.00	0.00	0.00
	N		0.00	3.31	0.03	0.00	0.00	1.57		0.00	6.46	0.16	0.00	0.00	8.24
2	R		0.00	11.86	0.00	0.00	0.00	0.00		0.00	30.11	0.00	0.00	0.00	0.00
	O		0.00	0.00	0.00	0.00	0.00	0.00		0.00	0.00	0.00	0.00	0.00	0.00
	N		0.20	0.00	0.00	0.16	0.10	1.19		1.37	0.00	0.00	1.56	0.00	8.40
3	R	4.17	0.28	0.00	0.00	0.14	0.00	1.63	25.24	2.32	0.00	0.00	1.49	0.00	8.47
	O	0.00	0.00	0.00	0.00	0.00	0.00	0.00	0.00	0.00	0.00	0.00	0.00	0.00	0.00
	N	6.24	0.18	0.01	0.01	0.20	0.12	0.78	16.28	0.09	0.00	0.00	1.06	0.01	3.00
4	R	0.00	0.00	0.19	0.00	0.00	3.79	0.00	0.00	0.00	0.10	0.00	0.00	29.74	0.00
	O	0.00	0.00	0.00	0.00	0.00	0.00	0.00	0.00	0.00	0.00	0.00	0.00	0.00	0.00
	N	4.62	1.68	0.00	0.00	0.25	0.03	0.01	2.04	0.25	0.00	0.00	1.19	0.03	0.00
5	R	0.00	1.93	0.00	0.01	1.60	14.77	0.00	0.00	3.24	0.00	0.01	2.62	5.57	0.00
	O	0.00	0.00	0.00	0.00	0.00	0.00	0.00	0.00	0.00	0.00	0.00	0.00	0.00	0.00
	N	3.31	0.30	0.00	0.00	0.93	0.54	0.06	1.55	0.03	0.00	0.00	0.22	0.19	0.00
6	R	0.00	0.00	0.00	0.00	0.00	0.00	0.00	0.00	0.00	0.00	0.00	0.00	0.00	0.00
	O	0.00	0.00	0.00	0.00	0.00	0.00	0.00	0.00	0.00	0.00	0.00	0.00	0.00	0.00
	N	1.22	0.48	0.97	0.00	0.25	0.37	0.75	1.42	4.23	0.03	0.00	0.03	0.23	10.32
7	R	25.08	28.70	20.85	16.33	20.28	30.89	33.06	48.39	66.07	42.00	82.98	68.96	35.38	62.11
	O	0.00	0.00	0.00	0.00	0.00	0.00	0.00	0.00	0.00	0.00	0.00	0.00	0.00	0.00
	N	0.22	1.65	0.12	0.08	0.06	0.19	0.01	0.16	3.94	0.55	31.01	10.76	0.29	9.69
8	R	0.51	2.87	0.90	0.35	0.22	0.19	1.03	0.22	5.76	0.98	34.79	12.71	0.69	10.08
	O	0.00	0.00	0.00	0.00	0.00	0.00	0.00	0.00	0.00	0.00	0.00	0.00	0.00	0.00

Sequence		GTP-triphosphate							GTP base+sugar						
		SGAGKT	GPSGSGKS	GKPNVGKS	GEPGTGKS	GPTGSGKS	GPESSGKT	GVNGVGKT	SGAGKT	GPSGSGKS	GKPNVGKS	GEPGTGKS	GPTGSGKS	GPESSGKT	GVNGVGKT
	**N**	68.86	75.47	69.85	80.01	77.66	68.97	83.31	27.19	51.15	44.83	77.26	65.40	23.86	49.28
**1**	**R**		0.00	0.00	0.00	0.00	0.00	0.00		0.00	0.00	0.00	0.00	0.00	0.00
	**O**		0.00	0.00	0.00	0.00	0.00	0.00		1.68	2.48	0.31	1.60	0.49	1.06
	**N**		0.00	28.91	0.81	0.00	0.00	24.58		0.00	4.25	0.73	0.00	0.00	1.66
**2**	**R**		0.00	42.90	0.00	0.00	0.00	0.00		0.00	3.81	0.61	0.00	0.00	0.00
	**O**		0.00	0.00	0.00	0.00	0.00	0.00		6.00	13.10	3.61	2.13	0.53	1.81
	**N**		4.82	0.00	0.00	2.06	0.14	26.35		3.08	0.00	0.00	0.84	0.43	1.04
**3**	**R**	37.05	7.30	0.00	0.00	2.14	0.00	23.48	1.22	1.18	0.00	0.00	1.25	3.14	3.06
	**O**	0.00	0.00	0.00	0.00	0.00	0.00	0.00	1.69	0.89	1.43	0.09	1.07	0.58	31.99
	**N**	40.16	2.32	0.02	0.01	1.43	0.16	13.95	8.84	8.27	15.85	26.86	18.61	0.71	2.90
**4**	**R**	0.00	0.00	1.77	0.00	0.00	45.34	0.00	0.00	0.00	3.41	0.00	0.00	1.58	0.00
	**O**	0.00	0.00	0.00	0.00	0.00	0.00	0.00	3.27	3.66	0.62	0.11	1.89	1.15	19.37
	**N**	10.77	4.80	0.08	0.01	4.33	0.14	1.88	2.80	6.70	16.39	33.67	20.00	0.79	2.16
**5**	**R**	0.00	12.80	0.00	0.14	12.49	37.49	0.00	0.00	4.53	0.00	5.34	3.54	2.00	0.00
	**O**	0.00	0.00	0.00	0.00	0.00	0.00	0.00	3.81	4.03	2.45	50.60	19.05	5.66	2.44
	**N**	7.87	8.66	0.01	0.32	6.19	1.05	2.39	3.21	15.47	13.29	24.24	25.20	2.32	4.40
**6**	**R**	0.00	0.00	0.00	0.00	0.00	0.00	0.00	0.00	0.00	0.00	0.00	0.00	0.00	0.00
	**O**	0.00	0.00	0.00	0.00	0.00	0.00	0.00	3.85	13.46	2.85	66.02	34.92	1.46	3.46
	**N**	2.86	14.54	5.10	0.43	3.03	2.32	13.48	5.34	2.93	3.67	1.65	4.30	2.87	15.29
**7**	**R**	68.39	80.03	56.55	90.81	82.19	71.95	77.50	12.56	14.74	6.74	37.85	25.25	27.17	5.47
	**O**	0.00	0.00	0.00	0.00	0.00	0.00	0.00	2.63	1.86	1.92	0.33	1.01	0.70	1.44
	**N**	0.83	11.31	3.64	31.67	11.85	2.09	10.35	5.11	2.08	1.83	0.28	1.49	1.70	3.94
**8**	**R**	1.38	16.44	5.91	35.79	14.44	3.01	11.93	5.61	3.87	3.17	0.80	2.19	2.41	4.85
	**O**	0.00	0.00	0.00	0.00	0.00	0.00	0.00	15.85	11.21	13.30	3.17	7.79	34.90	21.03

[a]Analysis based on snapshots taken every 10 ps of the simulation, calculated as described in the main text. The residue numbering of the SGAGKT hexapeptide has been shifted by +2 to align with the other peptides presented here for clarity. These data are shown visually in **Figs. 2** and **3**.

Supplementary Table 4: Peptide-ligand hydrogen bonding (% simulation time) between the peptides studied in this work, in complex with either HPO_4^{2-} or GTP.[a]

	HPO_4^{2-}	GTP P_α	GTP P_β	GTP P_γ	GTP triphosphate	GTP sugar+base	GTP whole
**SGAGKT**	36.50	76.50	48.98	79.23	97.30	70.21	99.20
**GPSGSGKS**	34.99	78.94	54.40	84.61	97.60	80.62	99.46
**GKPNVGKS**	39.91	79.48	51.36	83.48	97.82	77.28	99.56
**GEPGTGKS**	24.73	79.51	57.91	93.56	98.89	97.89	99.90
**GPTGSGKS**	30.83	77.34	55.00	87.20	98.03	87.20	99.61
**GPESSGKT**	27.63	75.37	57.09	79.60	96.60	74.36	98.77
**GVNGVGKT**	28.71	81.98	58.66	86.23	98.15	85.15	99.66
**KSPGGSSG**	49.14	84.15	43.89	88.54	99.29	77.57	99.81
**KGSGNKVP**	53.04	86.67	58.35	87.06	99.26	84.26	99.87
**EGKPGTSG**	25.17	73.71	43.70	72.72	94.69	76.11	98.32
**PGKGGSST**	38.57	79.26	52.08	78.30	97.28	66.89	99.18
**TGPSSEKG**	32.25	76.88	50.52	83.45	98.29	89.79	99.71
**VVTGGNKG**	25.48	80.85	47.55	72.05	95.98	75.51	98.89

[a] Analysis based on snapshots taken every 10 ps of the simulation, calculated as described in the main text. Sequences from GTP-binding NTPases are shown in green and sequences from ATP-binding NTPases are shown in blue.

Supplementary Table 5: Representative archaeal and bacterial NTPases studied in this work.[a]

Reference Domain	F-Group Name	Organism	%Identity (%Positives)	Strand Order	Walker A Sequence	Res. (Å)	Ligand
e2it1A8	ABC_tran	*Pyrococcus horikoshii*	47 (69)	23415(6)	GPSGSGKS	1.94	None (ATP)[b]
e3ievA2	MnmE_helical_2nd	*Aquifex aeolicus*	44 (61)	(2)31456	GKPNVGKS	1.9	GNP
e3k1jA2	Sigma54_activat	*Thermococcus onnurineus*	40 (54)	23415	GEPGTGKS	2	ADP
e6ojxA1	T2SSe	*Geobacter metallireducens*	40 (56)	324516(7)	GPTGSGKS	1.89	ATP
e5jrjA2	RecA	*Herbaspirillum seropedicae*	71 (85)	324516(7)8	GPESSGKT	1.7	ATP /ADP
e2qy9A2	SRP54	*Escherichia coli*	57 (72)	3241567	GVNGVGKT	1.9	None (GTP)[c]

[a] For details of how these NTPases were selected, see the main and **Supplementary Table 12**. %Identity and %Positives are relative to consensus hmmemit. For the strand order, ()=antiparallel. [b] While there is no ligand in the associated structure, Uniprot (The Uniprot Consortium 2018) lists the P-loop NTPase PH0203 from *Pyrococcus horikoshii* as a 362aa-long hypothetical maltose/maltodextrin ATP-binding protein. [c] While there is no ligand in the associated structure, the *E. coli* SRP-receptor FtsY (PDB ID: 2QY9 (Berman, et al. 2000)) has GTPase activity (Parlitz, et al. 2007).

Supplementary Table 6: Occurrence (% simulation time) of correlated stretches of α_L and α_R conformations in Walker A-derived octapeptides during simulations performed using the CHARMM36m force field (Huang, et al. 2016).

Sequence	4-5 (RL)	5-6 (RL)	6-7 (RL)	4-5 (LR)	5-6 (LR)	6-7 (LR)	4-6 (RLR)	5-7 (RLR)	4-6 (LRL)	5-7 (LRL)	4-7 (LRLR)
**Unliganded**											
**GPSGSGKS**	0.24	1.99	0.26	1.42	0.11	2.24	0.00	0.36	0.29	0.01	0.03
**GKPNVGKS**	0.05	1.24	0.25	1.73	0.00	0.93	0.00	0.11	0.33	0.00	0.07
**GEPGTGKS**	0.05	1.10	0.23	1.65	0.02	2.46	0.00	0.21	0.21	0.00	0.03
**GPTGSGKS**	0.34	1.16	0.24	1.29	0.12	2.36	0.00	0.25	0.15	0.00	0.03
**GPESSGKT**	0.45	1.01	0.51	0.88	0.14	1.29	0.02	0.21	0.06	0.03	0.01
**GVNGVGKT**	0.01	1.04	0.24	1.36	0.01	2.22	0.00	0.14	0.12	0.00	0.00

Supplementary Table 7: Occurrence (% simulation time) of correlated stretches of α_L and α_R conformations in Walker A-derived octapeptides in simulations with $C_{\alpha1}$-$C_{\alpha8}$ distance restraints.

Sequence	4-5 (RL)	5-6 (RL)	6-7 (RL)	4-5 (LR)	5-6 (LR)	6-7 (LR)	4-6 (RLR)	5-7 (RLR)	4-6 (LRL)	5-7 (LRL)	4-7 (LRLR)
**Unliganded**											
**GEPGTGKS**	0.43	9.88	0.97	12.17	0.09	8.79	0.00	2.50	2.52	0.00	0.65
**GPESSGKT**	7.40	23.37	0.46	1.94	0.59	23.24	0.10	9.68	0.74	0.00	0.34
**GVNGVGKT**	0.05	11.16	0.78	12.49	0.03	11.63	0.00	2.30	4.24	0.00	1.20
**HPO_4^{2-}**											
**GEPGTGKS**	0.14	9.56	1.23	13.48	0.09	9.10	0.01	2.84	2.42	0.03	0.76
**GPESSGKT**	6.85	20.39	0.35	1.68	0.68	23.07	0.24	9.00	0.85	0.04	0.41
**GVNGVGKT**	0.66	8.68	0.90	16.13	0.08	13.66	0.00	2.64	4.22	0.01	1.81
**GTP**											
**GEPGTGKS**	0.00	2.53	0.01	70.66	0.00	0.83	0.00	0.56	1.75	0.00	0.46
**GPESSGKT**	8.55	10.31	0.39	4.06	0.82	25.85	0.57	4.22	1.25	0.00	0.62
**GVNGVGKT**	0.39	2.10	0.19	18.79	0.08	4.74	0.06	0.97	1.15	0.00	0.58

Supplementary Table 8: Peptide-ligand hydrogen bonding (% simulation time) between the Walker A P-loop sequences studied in this work with $C_{\alpha 1}$-$C_{\alpha 8}$ distance restraints and either HPO_4^{2-} or GTP.[a]

		HPO_4^{2-}			GTP-α			GTP-β		
**Sequence**		GEPGTGKS	GPESSGKT	GVNGVGKT	GEPGTGKS	GPESSGKT	GVNGVGKT	GEPGTGKS	GPESSGKT	GVNGVGKT
	**N**	11.11	10.78	13.78	41.11	40.48	45.06	39.77	36.20	29.17
**1**	**R**	0.00	0.00	0.00	0.00	0.00	0.00	0.00	0.00	0.00
	**O**	0.09	0.07	0.09	0.00	0.00	0.00	0.00	0.00	0.00
	**N**	1.11	0.00	3.18	1.12	0.00	13.47	0.03	0.00	0.48
**2**	**R**	0.01	0.00	0.00	0.00	0.00	0.00	0.00	0.00	0.00
	**O**	0.10	0.01	0.06	0.00	0.00	0.00	0.00	0.00	0.00
	**N**	0.00	0.20	3.36	0.00	0.00	15.26	0.00	0.00	0.90
**3**	**R**	0.00	0.02	3.90	0.00	0.00	13.12	0.00	0.00	0.65
	**O**	0.00	0.00	0.07	0.00	0.00	0.00	0.00	0.00	0.00
	**N**	0.19	0.21	1.72	0.01	0.00	10.29	0.00	0.00	0.31
**4**	**R**	0.00	4.86	0.00	0.00	15.62	0.00	0.00	2.04	0.00
	**O**	0.01	0.01	0.01	0.00	0.00	0.00	0.00	0.00	0.00
	**N**	0.48	0.33	0.66	0.01	0.03	6.30	0.00	0.00	0.15
**5**	**R**	0.75	4.54	0.00	0.58	4.03	0.00	0.03	2.66	0.00
	**O**	0.01	0.01	0.00	0.00	0.00	0.00	0.00	0.00	0.00
	**N**	0.32	0.30	0.61	0.01	2.62	6.75	0.00	0.19	0.16
**6**	**R**	0.00	0.00	0.00	0.00	0.00	0.00	0.00	0.00	0.00
	**O**	0.02	0.01	0.02	0.00	0.00	0.00	0.00	0.00	0.00
	**N**	0.30	0.25	0.85	0.47	7.65	15.87	0.06	0.79	7.46
**7**	**R**	15.67	14.42	15.10	37.75	37.81	47.66	29.91	30.11	34.65
	**O**	0.01	0.00	0.01	0.00	0.00	0.00	0.00	0.00	0.00
	**N**	0.47	0.29	0.82	0.03	3.12	5.54	0.04	0.15	0.10
**8**	**R**	1.37	0.41	1.06	0.05	3.38	6.70	0.06	1.08	0.92
	**O**	0.01	0.00	0.01	0.00	0.00	0.00	0.00	0.00	0.00

Sequence		GTP-γ			GTP-triphosphate			GTP base+sugar		
		GEPGTGKS	GPESSGKT	GVNGVGKT	GEPGTGKS	GPESSGKT	GVNGVGKT	GEPGTGKS	GPESSGKT	GVNGVGKT
	**N**	51.80	36.19	53.10	81.67	76.05	81.63	80.15	43.27	47.25
**1**	**R**	0.00	0.00	0.00	0.00	0.00	0.00	0.00	0.00	0.00
	**O**	0.00	0.00	0.00	0.00	0.00	0.00	0.17	0.32	1.99
	**N**	0.16	0.00	7.98	1.31	0.00	21.83	0.45	0.00	1.42
**2**	**R**	0.00	0.00	0.00	0.00	0.00	0.00	0.17	0.00	0.00
	**O**	0.00	0.00	0.00	0.00	0.00	0.00	1.65	0.36	2.10
	**N**	0.00	0.04	8.05	0.00	0.05	23.96	0.00	1.19	1.68
**3**	**R**	0.00	0.00	8.25	0.00	0.00	21.35	0.00	1.85	4.67
	**O**	0.00	0.00	0.00	0.00	0.00	0.00	0.01	0.27	15.92
	**N**	0.00	0.08	2.17	0.01	0.09	12.50	15.69	2.11	12.62
**4**	**R**	0.00	12.82	0.00	0.00	30.31	0.00	0.00	2.06	0.00
	**O**	0.00	0.00	0.00	0.00	0.00	0.00	0.06	1.15	17.69
	**N**	0.00	0.01	0.00	0.01	0.04	6.45	32.33	0.88	5.61
**5**	**R**	0.07	5.40	0.00	0.68	11.89	0.00	6.87	2.97	0.00
	**O**	0.00	0.00	0.00	0.00	0.00	0.00	19.72	2.51	2.32
	**N**	0.00	0.01	0.00	0.01	2.77	6.82	47.38	2.81	10.76
**6**	**R**	0.00	0.00	0.00	0.00	0.00	0.00	0.00	0.00	0.00
	**O**	0.00	0.00	0.00	0.00	0.00	0.00	68.99	3.24	8.55
	**N**	0.05	0.45	1.07	0.58	8.76	20.25	2.39	1.67	10.28
**7**	**R**	82.19	49.26	64.87	91.94	67.37	82.01	46.77	5.94	9.18
	**O**	0.00	0.00	0.00	0.00	0.00	0.00	0.47	2.11	3.73
	**N**	1.24	0.42	1.05	1.30	3.67	6.68	0.39	2.90	7.23
**8**	**R**	1.54	0.54	1.26	1.65	4.75	8.81	1.26	4.25	7.92
	**O**	0.00	0.00	0.00	0.00	0.00	0.00	2.91	15.27	24.62

[a] Analysis based on snapshots taken every 10 ps of the simulation, calculated as described in the main text. These data are shown visually in **Supplementary Figure 7**.

Supplementary Table 9: Selected control sequences studied in this work

Reference Domain	X-Group Name	Organism	Control Seqeunce	Res. (Å)	Ligand
e1mt5A1	X-Group 7552	*Rattus norvegicus*	KSPGGSSG	2.30	None
e5dmyA4	Immunoglobulin-like beta-sandwich	*Bifidobacterium bifidum*	KGSGNKVP	1.95	None
e4bkrA1	Rossmann-like	*Yersinia pestis A1122*	EGKPGTSG	1.80	None
e1re5D1	L-Aspartase middle domain-like	*Pseudomonas putida KT2440*	PGKGGSST	2.60	None
e4lnbA1	Repetitive alpha hairpins	*Aspergillus fumigatus Af293*	TGPSSEKG	1.75	None
e3o26A1	Rossmann-like	*Papaver somniferum*	VVTGGNKG	1.91	NDP

Supplementary Table 10: Occurrence (% simulation time) of correlated stretches of α_L and α_R conformations in control octapeptides.

Sequence	4-5 (RL)	5-6 (RL)	6-7 (RL)	4-5 (LR)	5-6 (LR)	6-7 (LR)	4-6 (RLR)	5-7 (RLR)	4-6 (LRL)	5-7 (LRL)	4-7 (LRLR)
					**Unliganded**						
**KSPGGSSG**	1.23	0.94	3.75	1.07	5.05	3.83	0.51	0.35	0.26	0.18	0.05
**KGSGNKVP**	0.73	2.01	0.25	3.44	4.85	0.07	0.56	0.00	0.57	0.01	0.00
**EGKPGTSG**	0.40	0.17	6.50	0.00	19.13	1.23	0.21	0.04	0.00	2.74	0.00
**PGKGGSST**	0.49	0.64	8.83	0.54	4.70	3.94	0.12	0.24	0.05	0.70	0.02
**TGPSSEKG**	3.44	0.73	1.16	4.58	8.37	2.13	0.97	0.13	0.04	0.00	0.01
**VVTGGNKG**	0.76	0.70	1.08	1.38	3.91	2.31	0.11	0.13	0.09	0.23	0.01
					**HPO_4^{2-}**						
**KSPGGSSG**	1.03	0.81	2.34	1.21	5.79	4.27	0.24	0.37	0.03	0.30	0.01
**KGSGNKVP**	0.80	1.97	0.19	4.06	6.52	0.01	0.23	0.00	0.29	0.08	0.00
**EGKPGTSG**	0.39	0.13	6.11	0.00	17.87	0.79	0.18	0.03	0.00	2.52	0.00
**PGKGGSST**	0.02	1.22	1.50	0.09	0.53	9.65	0.00	0.49	0.03	0.07	0.02
**TGPSSEKG**	4.66	0.77	1.51	5.70	5.68	3.54	1.30	0.16	0.29	0.01	0.03
**VVTGGNKG**	0.78	0.34	0.91	1.56	3.86	1.08	0.12	0.12	0.03	0.07	0.01
					**GTP**						
**KSPGGSSG**	0.00	0.58	9.96	0.00	1.11	0.50	0.00	0.06	0.00	0.54	0.00
**KGSGNKVP**	7.04	0.56	0.00	0.02	4.33	0.07	1.18	0.00	0.00	0.00	0.00
**EGKPGTSG**	0.00	23.50	0.02	1.16	0.15	6.81	0.00	4.19	0.27	0.00	0.00
**PGKGGSST**	0.08	2.81	1.16	0.33	0.45	12.06	0.00	0.69	0.10	0.01	0.04
**TGPSSEKG**	5.54	18.19	0.37	2.96	0.29	10.67	0.29	2.86	1.18	0.24	0.07
**VVTGGNKG**	3.68	0.99	5.56	0.63	3.73	0.13	2.18	0.00	0.08	0.94	0.00

Supplementary Table 11: Peptide-ligand hydrogen bonding (% simulation time) between the control sequences studied in this work and either HPO_4^{2-} or GTP.[a]

Sequence		HPO_4^{2-}						GTP-α					
		KSPGGSSG	KGSGNKVP	EGKPGTSG	PGKGGSST	TGPSSEKG	VVTGGNKG	KSPGGSSG	KGSGNKVP	EGKPGTSG	PGKGGSST	TGPSSEKG	VVTGGNKG
	**N**	24.72	23.02	11.33	19.93	13.01	10.87	51.75	20.66	46.54	43.96	52.64	42.45
**1**	**R**	14.93	15.36	0.02	0.00	4.44	0.00	19.64	25.40	0.00	0.00	12.57	0.00
	**O**	0.11	0.11	0.12	0.21	0.10	0.06	0.00	0.00	0.00	0.00	0.00	0.00
	**N**	16.49	13.62	3.08	8.09	5.95	2.16	27.25	0.06	10.08	22.79	7.65	0.21
**2**	**R**	23.06	0.00	0.00	0.00	0.00	0.00	27.22	0.00	0.00	0.00	0.00	0.00
	**O**	0.05	0.12	0.09	0.22	0.14	0.01	0.00	0.00	0.00	0.00	0.00	0.00
	**N**	0.00	11.10	0.40	1.73	0.00	3.65	0.00	39.27	3.80	12.82	0.00	3.77
**3**	**R**	0.00	11.76	12.41	15.88	0.00	3.60	0.00	35.69	34.86	40.75	0.00	24.59
	**O**	0.00	0.02	0.02	0.05	0.00	0.03	0.00	0.00	0.00	0.00	0.00	0.00
	**N**	0.14	3.20	0.00	0.73	0.47	1.83	0.01	8.42	0.00	5.09	0.17	3.14
**4**	**R**	0.00	0.00	0.00	0.00	2.73	0.00	0.00	0.00	0.00	0.00	2.55	0.00
	**O**	0.01	0.06	0.00	0.04	0.01	0.03	0.00	0.00	0.00	0.00	0.00	0.00
	**N**	0.09	0.40	0.09	0.24	0.39	0.45	0.87	2.80	0.03	1.44	0.61	3.95
**5**	**R**	0.00	1.83	0.00	0.00	3.84	0.00	0.00	3.49	0.00	0.00	8.65	0.00
	**O**	0.13	0.02	0.00	0.01	0.01	0.01	0.00	0.00	0.00	0.00	0.00	0.00
	**N**	2.60	0.51	0.56	1.85	0.05	0.35	1.87	3.27	0.05	2.73	0.26	1.92
**6**	**R**	6.13	14.50	0.90	4.28	0.01	0.99	2.16	24.78	0.98	2.90	0.00	6.08
	**O**	0.02	0.00	0.01	0.01	0.01	0.01	0.00	0.00	0.00	0.00	0.00	0.00
	**N**	2.90	0.08	0.62	1.68	0.03	0.13	1.26	0.01	0.04	2.14	1.87	2.07
**7**	**R**	5.68	0.00	2.49	3.34	14.76	14.47	2.73	0.00	1.06	3.37	29.03	49.52
	**O**	0.01	0.01	0.01	0.00	0.01	0.00	0.00	0.00	0.00	0.00	0.00	0.00
	**N**	0.43	0.00	0.14	0.66	0.07	0.03	0.49	0.00	0.24	0.09	1.84	0.20
**8**	**R**	0.00	0.00	0.00	0.89	0.00	0.00	0.00	0.00	0.00	0.31	0.00	0.00
	**O**	0.02	0.01	0.01	0.01	0.01	0.01	0.00	0.00	0.00	0.00	0.00	0.00

Sequence			KSPGGSSG	KGSGNKVP	EGKPGTSG	PGKGGSST	TGPSSEKG	VVTGGNKG		KSPGGSSG	KGSGNKVP	EGKPGTSG	PGKGGSST	TGPSSEKG	VVTGGNKG
	**N**	GTP-β	20.49	17.22	26.01	18.44	24.45	20.95	GTP-γ	67.04	43.86	47.28	39.13	45.74	23.56
**1**	**R**		20.86	27.55	0.00	0.00	2.70	0.00		33.01	46.30	0.00	0.00	20.03	0.00
	**O**		0.00	0.00	0.00	0.00	0.00	0.00		0.00	0.00	0.00	0.00	0.00	0.00
	**N**		6.46	0.08	3.25	10.83	4.49	0.17		35.74	0.01	15.53	14.89	27.04	0.05
**2**	**R**		5.49	0.00	0.00	0.00	0.00	0.00		40.42	0.00	0.00	0.00	0.00	0.00
	**O**		0.00	0.00	0.00	0.00	0.00	0.00		0.00	0.00	0.00	0.00	0.00	0.00
	**N**		0.00	7.05	1.31	8.42	0.00	0.92		0.00	6.17	3.85	2.98	0.00	0.93
**3**	**R**		0.00	8.27	21.69	33.44	0.00	7.58		0.00	7.86	38.21	51.76	0.00	2.93
	**O**		0.00	0.00	0.00	0.00	0.00	0.00		0.00	0.00	0.00	0.00	0.00	0.00
	**N**		0.00	0.40	0.00	1.36	0.00	0.27		0.00	0.47	0.00	0.48	1.52	0.60
**4**	**R**		0.00	0.00	0.00	0.00	0.82	0.00		0.00	0.00	0.00	0.00	5.21	0.00
	**O**		0.00	0.00	0.00	0.00	0.00	0.00		0.00	0.00	0.00	0.00	0.00	0.00
	**N**		0.06	0.26	0.00	0.10	0.05	0.66		0.01	0.05	0.01	0.22	1.21	0.51
**5**	**R**		0.00	0.69	0.00	0.00	2.64	0.00		0.00	1.04	0.00	0.00	3.34	0.00
	**O**		0.00	0.00	0.00	0.00	0.00	0.00		0.00	0.00	0.00	0.00	0.00	0.00
	**N**		0.07	0.07	0.00	0.46	0.02	0.03		0.08	0.22	0.00	0.73	0.04	0.04
**6**	**R**		0.13	19.21	5.04	0.86	0.00	1.39		0.97	31.80	1.93	2.54	0.00	0.73
	**O**		0.00	0.00	0.00	0.00	0.00	0.00		0.00	0.00	0.00	0.00	0.00	0.00
	**N**		0.01	0.00	0.00	0.42	0.00	0.54		0.08	0.00	0.16	1.82	0.00	0.00
**7**	**R**		0.43	0.00	0.20	1.01	25.73	26.82		1.67	0.00	0.64	3.06	58.01	58.74
	**O**		0.00	0.00	0.00	0.00	0.00	0.00		0.00	0.00	0.00	0.00	0.00	0.00
	**N**		0.02	0.00	0.17	0.01	0.37	0.00		0.01	0.00	0.03	0.00	0.00	0.00
**8**	**R**		0.00	0.00	0.00	0.10	0.00	0.00		0.00	0.00	0.00	0.06	0.00	0.00
	**O**		0.00	0.00	0.00	0.00	0.00	0.00		0.00	0.00	0.00	0.00	0.00	0.00

Sequence		GTP-triphosphate						GTP base+sugar					
		KSPGGSSG	KGSGNKVP	EGKPGTSG	PGKGGSST	TGPSSEKG	VVTGGNKG	KSPGGSSG	KGSGNKVP	EGKPGTSG	PGKGGSST	TGPSSEKG	VVTGGNKG
	**N**	86.08	63.24	74.53	73.00	79.28	62.33	41.68	15.59	21.29	7.79	63.99	29.68
**1**	**R**	50.55	62.57	0.00	0.00	34.98	0.00	2.01	3.24	3.10	0.00	0.19	0.00
	**O**	0.00	0.00	0.00	0.00	0.00	0.00	1.29	0.50	3.25	3.39	1.06	1.20
	**N**	67.65	0.14	27.88	42.52	38.74	0.42	6.93	3.92	8.64	6.40	1.58	2.22
**2**	**R**	72.37	0.00	0.00	0.00	0.00	0.00	3.81	0.00	0.00	0.00	0.00	0.00
	**O**	0.00	0.00	0.00	0.00	0.00	0.00	4.75	8.17	4.60	5.36	0.47	4.17
	**N**	0.00	51.15	8.90	23.29	0.00	5.57	0.00	3.69	5.03	4.69	0.00	0.82
**3**	**R**	0.00	51.68	58.74	72.46	0.00	34.66	0.00	3.90	20.19	9.75	0.00	3.44
	**O**	0.00	0.00	0.00	0.00	0.00	0.00	2.31	1.92	5.33	3.00	2.03	2.77
	**N**	0.01	9.18	0.00	5.89	1.69	3.97	1.38	13.52	0.00	3.48	6.47	6.29
**4**	**R**	0.00	0.00	0.00	0.00	8.50	0.00	0.00	0.00	0.00	0.00	0.63	0.00
	**O**	0.00	0.00	0.00	0.00	0.00	0.00	8.07	14.51	7.42	4.18	16.68	7.36
	**N**	0.94	2.94	0.03	1.71	1.86	4.69	4.60	7.42	1.32	6.77	21.86	4.16
**5**	**R**	0.00	4.95	0.00	0.00	14.44	0.00	0.00	4.56	0.00	0.00	8.14	0.00
	**O**	0.00	0.00	0.00	0.00	0.00	0.00	5.68	11.40	6.50	6.82	0.77	12.34
	**N**	1.98	3.54	0.05	3.79	0.32	1.99	5.10	7.29	1.03	8.59	38.85	4.12
**6**	**R**	3.23	50.28	7.71	6.21	0.00	7.75	2.90	6.78	4.10	3.84	20.64	8.12
	**O**	0.00	0.00	0.00	0.00	0.00	0.00	1.67	4.03	1.48	2.46	1.84	4.66
	**N**	1.34	0.01	0.21	4.14	1.87	2.61	6.22	1.48	1.30	8.53	1.54	4.83
**7**	**R**	4.80	0.00	1.88	7.28	71.57	76.88	4.31	0.00	2.94	4.16	3.62	11.06
	**O**	0.00	0.00	0.00	0.00	0.00	0.00	1.84	2.56	3.18	4.43	1.25	2.38
	**N**	0.51	0.00	0.39	0.09	2.17	0.21	5.24	0.00	5.45	2.35	1.62	2.81
**8**	**R**	0.00	0.00	0.00	0.47	0.00	0.00	0.00	0.00	0.00	5.57	0.00	0.00
	**O**	0.00	0.00	0.00	0.00	0.00	0.00	17.33	33.95	22.91	14.70	44.40	19.44

[a] Analysis based on snapshots taken every 10 ps of the simulation, calculated as described in the main text. These data are shown visually in **Supplementary Figure 9**.

Supplementary Table 12: Occurrence (% simulation time) of correlated stretches of α_L and α_R conformations in simulations of the SGAGKT, GPSGSGKS, and KSPGGSSG peptides in methanol.

**Sequence**	**4-5 (RL)**	**5-6 (RL)**	**6-7 (RL)**	**4-5 (LR)**	**5-6 (LR)**	**6-7 (LR)**	**4-6 (RLR)**	**5-7 (RLR)**	**4-6 (LRL)**	**5-7 (LRL)**	**4-7 (LRLR)**
**Unliganded**											
**SGAGKT**	0.09	1.52	2.72	0.47	0.05	10.17	0.00	0.42	0.06	0.00	0.03
**GPSGSGKS**	0.17	4.96	0.76	3.85	0.34	2.38	0.00	1.45	2.00	0.00	0.32
**KSPGGSSG**	1.03	0.78	1.83	0.77	29.75	2.36	0.66	0.39	0.11	0.30	0.02
**HPO_4^{2-}**											
**SGAGKT**	0.16	1.73	0.26	3.82	0.20	15.48	0.05	0.53	0.81	0.00	0.19
**GPSGSGKS**	0.14	40.69	0.28	39.75	0.03	39.06	0.00	35.69	36.77	0.01	34.27
**KSPGGSSG**	4.09	0.13	1.74	3.61	8.15	2.12	0.03	0.08	0.05	0.20	0.04
**GTP**											
**SGAGKT**	0.43	6.19	1.52	7.94	0.29	10.29	0.01	0.96	1.21	0.00	0.61
**GPSGSGKS**	0.03	44.90	0.51	34.47	0.00	5.18	0.00	2.08	32.38	0.00	1.48
**KSPGGSSG**	0.00	0.45	0.45	0.05	3.18	8.36	0.00	0.26	0.04	0.10	0.01

Supplementary Table 13. Peptide-ligand hydrogen bonding and ionic interactions (% simulation time) during simulations of the SGAGKT, GPSGSGKS, and KSPGGSSG peptides, with either HPO_4^{2-} or GTP.[a]

		HPO_4^{2-}			GTP-α			GTP-β		
**Sequence**		SGAGKT	GPSGSGKS	KSPGGSSG	SGAGKT	GPSGSGKS	KSPGGSSG	SGAGKT	GPSGSGKS	KSPGGSSG
	**N**	58.29	59.58	66.18	33.64	40.94	36.99	26.72	25.56	46.74
**1**	**R**	0.00	0.00	50.40	0.00	0.00	55.91	0.00	0.00	41.17
	**O**	0.00	0.13	0.02	0.00	0.00	0.00	0.00	0.00	0.00
	**N**	0.00	0.00	56.21	0.00	0.00	2.91	0.00	0.00	3.93
**2**	**R**	0.00	0.00	62.75	0.00	0.00	5.62	0.00	0.00	2.72
	**O**	0.00	0.02	0.27	0.00	0.00	0.00	0.00	0.00	0.00
	**N**	0.00	44.24	0.00	0.00	2.17	0.00	0.00	0.62	0.00
**3**	**R**	24.86	46.07	0.00	8.54	2.30	0.00	10.82	1.90	0.00
	**O**	0.10	0.04	0.00	0.00	0.00	0.00	0.00	0.00	0.00
	**N**	44.78	33.06	0.64	20.90	1.60	0.06	11.90	1.11	0.00
**4**	**R**	0.00	0.00	0.00	0.00	0.00	0.00	0.00	0.00	0.00
	**O**	0.19	0.13	0.00	0.00	0.00	0.00	0.00	0.00	0.00
	**N**	19.36	42.40	0.15	3.36	1.19	2.07	11.53	2.68	0.69
**5**	**R**	0.00	53.91	0.00	0.00	3.10	0.00	0.00	14.37	0.00
	**O**	0.18	0.13	0.43	0.00	0.00	0.00	0.00	0.00	0.00
	**N**	12.16	33.45	24.05	5.75	3.56	3.05	8.19	1.92	2.40
**6**	**R**	0.00	0.00	42.23	0.00	0.00	8.24	0.00	0.00	5.47
	**O**	0.10	0.15	0.11	0.00	0.00	0.00	0.00	0.00	0.00
	**N**	9.52	47.30	28.95	0.66	1.71	7.15	6.56	1.83	4.78
**7**	**R**	82.30	85.29	39.15	45.44	47.66	4.32	50.63	39.68	5.91
	**O**	0.24	0.46	0.32	0.00	0.00	0.00	0.00	0.00	0.00
	**N**	3.54	40.24	10.03	0.45	1.31	0.00	2.28	1.27	0.13
**8**	**R**	4.19	43.37	0.00	0.48	1.43	0.00	1.57	2.77	0.00
	**O**	0.31	1.14	1.09	0.00	0.00	0.00	0.00	0.00	0.00

**Sequence**	SGAGKT	GPSGSGKS	KSPGGSSG	SGAGKT	GPSGSGKS	KSPGGSSG	SGAGKT	GPSGSGKS	KSPGGSSG

		GTP-γ			GTP-triphosphate			GTP base+sugar		
	**N**	40.11	26.56	42.23	77.71	73.76	85.89	22.92	28.70	9.19
**1**	**R**	0.00	0.00	35.85	0.00	0.00	88.56	0.00	0.00	34.83
	**O**	0.00	0.00	0.00	0.00	0.00	0.00	0.00	0.79	0.71
	**N**	0.00	0.00	19.98	0.00	0.00	26.42	0.00	0.00	0.19
**2**	**R**	0.00	0.00	19.18	0.00	0.00	27.44	0.00	0.00	0.81
	**O**	0.00	0.00	0.00	0.00	0.00	0.00	0.00	0.85	0.40
	**N**	0.00	16.53	0.00	0.00	19.24	0.00	0.00	8.97	0.00
**3**	**R**	22.41	48.92	0.00	41.59	52.47	0.00	1.64	0.52	0.00
	**O**	0.00	0.00	0.00	0.00	0.00	0.00	1.11	1.28	1.51
	**N**	23.70	3.33	0.02	53.59	5.62	0.08	2.87	3.53	0.48
**4**	**R**	0.00	0.00	0.00	0.00	0.00	0.00	0.00	0.00	0.00
	**O**	0.00	0.00	0.00	0.00	0.00	0.00	3.45	0.44	0.36
	**N**	13.10	2.62	37.83	27.47	5.78	40.38	0.33	1.39	1.00
**5**	**R**	0.00	35.69	0.00	0.00	53.05	0.00	0.00	3.56	0.00
	**O**	0.00	0.00	0.00	0.00	0.00	0.00	2.14	2.88	0.49
	**N**	12.48	0.02	39.94	22.62	4.13	44.97	2.13	1.47	1.05
**6**	**R**	0.00	0.00	43.53	0.00	0.00	56.99	0.00	0.00	2.22
	**O**	0.00	0.00	0.00	0.00	0.00	0.00	2.17	0.11	0.49
	**N**	12.34	0.03	17.61	19.46	3.37	25.91	4.13	2.21	0.47
**7**	**R**	74.34	75.45	21.28	96.34	97.59	30.97	7.05	2.13	2.23
	**O**	0.00	0.00	0.00	0.00	0.00	0.00	3.12	0.99	1.54
	**N**	8.89	0.00	0.25	10.72	2.57	0.38	6.73	0.84	0.58
**8**	**R**	11.07	2.50	0.00	11.94	6.68	0.00	2.68	1.17	0.00
	**O**	0.00	0.00	0.00	0.00	0.00	0.00	33.20	40.03	29.13

[a] Analysis based on snapshots taken every 10 ps of the simulation, calculated as described in the main text. This data is shown visually in **Figure 5**.

Supplementary Table 14: Phyletic distribution of P-loop NTPase families across the microbial tree of life.[a]

F-Group Name	Frac. Species, Archaea	Frac. Species, Bacteria	Frac. Phyla, Archaea	Frac. Phyla, Bacteria
AAA_31	0.95	0.96	1.00	1.00
ABC_tran	1.00	1.00	1.00	1.00
ABC_tran_1	1.00	1.00	1.00	1.00
DEAD	1.00	1.00	1.00	1.00
DEAD_1	1.00	1.00	1.00	1.00
MnmE_helical_2nd	1.00	1.00	1.00	1.00
Sigma54_activat	1.00	1.00	1.00	1.00
AAA_31_1	0.93	0.95	1.00	0.99
DEAD_3	1.00	0.99	1.00	0.99
T2SSE	0.93	0.80	1.00	0.99
SMC_N	0.77	0.96	1.00	0.98
RecA	0.93	0.93	1.00	0.98
Helicase_C_1	0.97	0.96	1.00	0.96
CTP_synth_N	0.85	0.90	1.00	0.95
SRP54	0.97	0.93	1.00	0.91
Helicase_C	0.53	0.51	1.00	0.83
Beta-Casp	0.96	0.34	1.00	0.70
DUF853	0.69	0.36	1.00	0.65
AAA	0.96	0.07	1.00	0.48
Ras_1	0.75	0.99	0.94	0.98
Thymidylate_kin	0.89	0.88	0.94	0.98
SMC_N_1	0.57	0.68	0.94	0.88
Helicase_C_2	0.82	0.53	0.94	0.77
DEAD_2	0.74	0.46	0.94	0.76
Cytidylate_kin2	0.75	0.21	0.94	0.65
Helicase_C_5	0.85	0.28	0.94	0.58
MCM_AAA	0.69	0.01	0.94	0.27
POR	0.88	0.64	0.89	0.98
Ras	0.67	0.98	0.89	0.97
ATP-synt_ab	0.83	0.97	0.83	1.00
CobA_CobO_BtuR	0.41	0.43	0.83	0.79
sufC	0.55	0.64	0.83	0.60
SKI	0.36	0.89	0.78	0.93
UvrD_C	0.36	0.97	0.72	0.98
SNF2_N	0.49	0.66	0.72	0.85
AAA_33	0.29	0.50	0.72	0.69

DEAD_4	0.23	0.26	0.72	0.31
ResIII	0.51	0.94	0.67	0.98
NTPase_1	0.44	0.04	0.67	0.53
NB-ARC_1st	0.25	0.08	0.67	0.37
Mur_ligase_M	0.43	0.99	0.61	1.00
Helicase_C_6	0.29	0.31	0.61	0.82
AAA_19	0.23	0.74	0.61	0.78
dNK_2	0.09	0.29	0.61	0.60
AAA_12	0.50	0.34	0.61	0.58
PEPCK_C	0.11	0.25	0.61	0.54
PEPCK_N	0.11	0.25	0.61	0.53
ATPase_2	0.16	0.05	0.61	0.42
AAA_25	0.11	0.97	0.56	0.98
MutS_V	0.44	0.78	0.56	0.95
Adenylsucc_synt	0.46	0.87	0.56	0.93
PEPCK_ATP_C	0.26	0.45	0.56	0.74
APS_kinase	0.25	0.46	0.56	0.73
PRK	0.20	0.66	0.56	0.73
APS_kinase_1	0.26	0.46	0.56	0.72
Sulfotransfer_1	0.09	0.26	0.56	0.65
TK_N	0.14	0.49	0.56	0.48
MG423	0.48	0.58	0.56	0.44
SUA5	0.18	0.19	0.56	0.29
Bac_DnaA_N	0.12	0.96	0.50	0.98
DnaB_C	0.03	0.94	0.50	0.98
UvrD_C_2	0.04	0.79	0.50	0.89
UvrD-helicase	0.24	0.70	0.50	0.79
dNK	0.05	0.27	0.50	0.58
Terminase_6	0.09	0.10	0.50	0.53
cobW	0.32	0.51	0.50	0.45
AAA_11	0.22	0.11	0.50	0.44
Helicase_RecD	0.30	0.06	0.50	0.04
DUF1726	0.28	0.04	0.50	0.02
CoaE	0.02	0.90	0.44	0.98
Torsin	0.06	0.95	0.44	0.97
FtsK_SpoIIIE	0.02	0.91	0.44	0.92
FTHFS	0.26	0.40	0.44	0.70
AAA_26	0.36	0.56	0.44	0.68
MobB	0.32	0.25	0.44	0.60

COG5410	0.03	0.04	0.44	0.38
Guanylate_kin	0.02	0.92	0.39	0.96
DUF1846_N	0.02	0.12	0.39	0.31
PhoH	0.06	0.86	0.33	0.91
Sigma54_activ_2	0.00	0.41	0.33	0.82
ArgK	0.46	0.40	0.33	0.76
RsgA_GTPase	0.06	0.73	0.33	0.67
PEPCK_ATP_N	0.16	0.42	0.33	0.66
AAA_32_2nd	0.07	0.16	0.33	0.56
SMC_N_2	0.02	0.15	0.33	0.56
Helicase_C_8	0.06	0.21	0.33	0.53
ABC_ATPase_C	0.06	0.07	0.33	0.47
DUF1611	0.28	0.10	0.33	0.33
Terminase_3	0.01	0.08	0.33	0.26
CPT	0.01	0.06	0.33	0.24
CLP1_P	0.11	0.00	0.33	0.23
Helicase_C_10	0.00	0.93	0.28	0.95
SecA_DEAD	0.00	0.92	0.28	0.95
dNK_1	0.03	0.26	0.28	0.56
CobU	0.01	0.43	0.28	0.53
PPK2	0.13	0.52	0.28	0.52
Sulfotransfer_2	0.01	0.03	0.28	0.24
Gtr1_RagA_N	0.05	0.01	0.28	0.12
AAA_28	0.01	0.17	0.22	0.24
UvrD_C_2_1	0.00	0.06	0.22	0.18
Thymidylate_kin_1	0.01	0.00	0.22	0.08
GTP_HydF_2nd	0.00	0.11	0.17	0.52
GTP_HydF_1st	0.00	0.11	0.17	0.50
KdpD	0.03	0.32	0.17	0.40
TrwB_AAD_bind	0.05	0.27	0.17	0.38
AAA_11_1	0.01	0.05	0.17	0.27
NACHT	0.00	0.02	0.17	0.19
6PF2K	0.00	0.01	0.17	0.12
TsaE	0.00	0.92	0.11	0.96
Cytidylate_kin_1	0.00	0.86	0.11	0.94
Cytidylate_kin_2	0.00	0.86	0.11	0.94
Cytidylate_kin_3	0.00	0.86	0.11	0.94
Cytidylate_kin_4	0.00	0.86	0.11	0.94
MlrC_C	0.03	0.12	0.11	0.31

CE15	0.00	0.06	0.11	0.24
DUF927_C	0.01	0.06	0.11	0.24
Sulfotransfer_3	0.00	0.04	0.11	0.19
AAA_6_N	0.00	0.00	0.11	0.07
Podovirus_Gp16	0.00	0.00	0.11	0.05
AAA_7_N	0.01	0.00	0.11	0.02
Helicase_C_9	0.00	0.80	0.06	0.91
LpxK_N	0.00	0.49	0.06	0.74
Hpr_kinase_C	0.00	0.35	0.06	0.62
Kinase-PPPase_C	0.00	0.36	0.06	0.40
Kinase-PPPase_N	0.00	0.15	0.06	0.37
Zeta_toxin	0.00	0.03	0.06	0.16
Dynamin_N	0.00	0.00	0.06	0.09
AAA_5	0.00	0.00	0.06	0.07
P-mevalo_kinase	0.00	0.01	0.06	0.06
Viral_helicase1	0.00	0.00	0.06	0.05
AAA_8	0.00	0.00	0.06	0.03
P-mevalo_kinase_1	0.00	0.00	0.06	0.03
AAA_9_N	0.00	0.00	0.06	0.02
Dynein_heavy_1st	0.00	0.00	0.06	0.02
AAA_22_like	0.00	0.00	0.06	0.01
DUF257	0.03	0.00	0.06	0.00
TIP49_1st	0.00	0.00	0.06	0.00
DNA_pol3_delta_N	0.00	0.62	0.00	0.76
UvrD-helicase_1	0.00	0.17	0.00	0.30
Exonuc_V_gamma	0.00	0.19	0.00	0.25
Helicase_C_7	0.00	0.10	0.00	0.21
Sulphotransf	0.00	0.03	0.00	0.15
Zot	0.00	0.02	0.00	0.15
DNA_pol3_chi	0.00	0.27	0.00	0.11
IPT	0.00	0.01	0.00	0.09
Cas_Csn2	0.00	0.02	0.00	0.08
MukB	0.00	0.03	0.00	0.08
HDA2-3	0.00	0.00	0.00	0.04
Septin	0.00	0.00	0.00	0.04
Cas_St_Csn2	0.00	0.00	0.00	0.03
Kinesin	0.00	0.00	0.00	0.02
Myosin_head	0.00	0.00	0.00	0.02

Myosin_head_1	0.00	0.00	0.00	0.02
Myosin_head_2	0.00	0.00	0.00	0.02
Parvo_NS1	0.00	0.00	0.00	0.02
PRK10689	0.00	0.10	0.00	0.02
PRK13709	0.00	0.00	0.00	0.02
SulA	0.00	0.08	0.00	0.02
DENN	0.00	0.00	0.00	0.01
Herpes_TK	0.00	0.00	0.00	0.01
PRK07993_1st	0.00	0.01	0.00	0.01
RHD3_1st	0.00	0.00	0.00	0.01
AAA_33_like	0.00	0.00	0.00	0.00
AAA_33,ADK_lid	0.00	0.00	0.00	0.00
AAA_33,RNA_pol_Rpc34	0.00	0.00	0.00	0.00
ABC_tran_3	0.00	0.00	0.00	0.00
Adaptin_binding	0.00	0.00	0.00	0.00
ADK_lid,Cytidylate_kin_2	0.00	0.00	0.00	0.00
ATP-synt_ab,ATP-synt_ab_C	0.00	0.00	0.00	0.00
ATP-synt_ab,ATP-synt_ab_N	0.00	0.00	0.00	0.00
CDC73_C	0.00	0.00	0.00	0.00
CENP-M	0.00	0.00	0.00	0.00
COG5177	0.00	0.00	0.00	0.00
CSM2	0.00	0.00	0.00	0.00
Cytidylate_kin_2,Thymidylate_kin	0.00	0.00	0.00	0.00
DAP3_C,DAP3_N	0.00	0.00	0.00	0.00
DAP3_N	0.00	0.00	0.00	0.00
DEAD_1,KOG0951_2nd	0.00	0.00	0.00	0.00
DLIC	0.00	0.00	0.00	0.00
Elong_Iki1	0.00	0.00	0.00	0.00
ELP6	0.00	0.00	0.00	0.00
EUF08601	0.00	0.00	0.00	0.00
F_UNCLASSIFIED	0.00	0.00	0.00	0.00
Flavi_DEAD	0.00	0.00	0.00	0.00
Folliculin_C	0.00	0.00	0.00	0.00
GBP	0.00	0.00	0.00	0.00
GBP_1	0.00	0.00	0.00	0.00
HA2_N,DEAD_3	0.00	0.00	0.00	0.00
IPPT,IPT	0.00	0.00	0.00	0.00

KOG0163_1,Myosin_head_1	0.00	0.00	0.00	0.00
KOG0384_1,Helicase_C	0.00	0.00	0.00	0.00
KOG2538_1st	0.00	0.00	0.00	0.00
LAP1C	0.00	0.00	0.00	0.00
Microtub_bd	0.00	0.00	0.00	0.00
MobB_1	0.00	0.00	0.00	0.00
Myosin_head,IQ_2	0.00	0.00	0.00	0.00
NTPase_P4	0.00	0.00	0.00	0.00
Orbi_VP4_1st	0.00	0.00	0.00	0.00
ORC2_1st	0.00	0.00	0.00	0.00
ORC3_N_1st	0.00	0.00	0.00	0.00
PAXNEB	0.00	0.00	0.00	0.00
Polyoma_lg_T_C	0.00	0.00	0.00	0.00
PRK11000,ABC_tran	0.00	0.00	0.00	0.00
PRK11000,ABC_tran_1	0.00	0.00	0.00	0.00
PSY3	0.00	0.00	0.00	0.00
Rap_GAP	0.00	0.00	0.00	0.00
Sigma54_activat,CDC48	0.00	0.00	0.00	0.00
Sigma54_activat,KOG0729	0.00	0.00	0.00	0.00
Sigma54_activat,Vps4_C_C	0.00	0.00	0.00	0.00
Torsin,RecA	0.00	0.00	0.00	0.00
UNK_F_TYPE	0.00	0.00	0.00	0.00
UvrA,ABC_tran	0.00	0.00	0.00	0.00

[a] Distributions were calculated as described in the main text. The seven representative families chosen for further analysis are written in red, with the selection criteria provided in the main text.

Supplementary Table 15: The ff99SB-ILDN (Lindorff-Larsen, et al. 2010) force field parameters used to describe the ligand HPO_4^{2-}.

Atom Name	Atom Type	Partial Charge	Atom Name	Atom Type	Partial Charge
P	PP	1.320903	O3	O2P	-0.956501
O1	OHP	-0.815401	O4	O2P	-0.956501
O2	O2P	-0.956501	HOP	HOP	0.364000

Supplementary Table 16: The ff99SB-ILDN (Lindorff-Larsen, et al. 2010) force field parameters used to describe the ligand GTP.

Atom Name	Atom Type	Partial Charge	Atom Name	Atom Type	Partial Charge
O1G	O3	-0.9526	C8	CK	0.1374
PG	P	1.2650	H8	H5	0.1640
O2G	O3	-0.9526	N7	NB	-0.5709
O3G	O3	-0.9526	C5	CB	0.1744
O3B	OS	-0.5322	C6	C	0.4770
PB	P	1.3852	O6	O	-0.5597
O1B	O2	-0.8894	N1	NA	-0.4787
O2B	O2	-0.8894	H1	H	0.3424
O3A	OS	-0.5689	C2	CA	0.7657
PA	P	1.2532	N2	N2	-0.9672
O1A	O2	-0.8799	H21	H	0.4364
O2A	O2	-0.8799	H22	H	0.4364
O5'	OS	-0.5987	N3	NC	-0.6323
C5'	CT	0.0558	C4	CB	0.1222
H5'1	H1	0.0679	C3'	CT	0.2022
H5'2	H1	0.0679	O3'	OH	-0.6541
C4'	CT	0.1065	H3T	HO	0.4376
H4'	H1	0.1174	H3'	H1	0.0615
O4'	OS	-0.3548	C2'	CT	0.0670
C1'	CT	0.0191	H2'1	H1	0.0972
H1'	H2	0.2006	O2'	OH	-0.6139
N9	N*	0.0492	HO'2	HO	0.4186

Supplementary Table 17: List of distance restraints between the $C_{\alpha 1}$ and $C_{\alpha 8}$ atoms of the peptides studied in the restrained simulations.[a]

Sequence	r_0, nm	r_1, nm	r_2, nm	Force constant, kJ mol^{-1} nm^{-1}
GEPGTGKS	1.00	1.11	1.20	4184
GPESSGKT	0.94	01.04	1.14	4184
GVNGVGKT	0.94	01.04	1.14	4184

[a] A piecewise linear-harmonic restraint potential was used (bond type 10 in GROMACS (van der Spoel, et al. 2005)). The potential is quadratic for $r_{ij} \leq r_0$, $r_0 \leq r_{ij} \leq r_1$ and $r_1 \leq r_{ij} \leq r_2$, and linear for $r_2 \leq r_{ij}$.

Supplementary References

Berman HM, Westbrook J, Feng Z, Gilliland G, Bhat TN, Weissig H, Shindyalov IN, Bourne PE. 2000. The Protein Data Bank. Nucleic Acids Res. 28:235-242.

Bussi G, Donadio D, Parrinello M. 2007. Canonical Sampling Through Velocity Rescaling. J. Chem. Phys. 126:014101.

Caldwell JW, Kollmann PA. 1995. Structure and Properties of Neat Liquids Using Nonadditive Molecular Dynamics: Water, Methanol, and N-Methylacetamide. J. Phys. Chem. 99:6208-6219.

Darden T, York D, Pedersen L. 1993. Particle Mesh Ewald: An *N*·log(*N*) Method for Ewald Sums in Large Systems. J. Chem. Phys. 98:10089-10092.

Hess B. 2008. P-LINCS: A Parallel Linear Constraint Solver for Molecular Simulation. J. Chem. Theory Comput. 4:116-122.

Huang J, Rauscher S, Nawrocki G, Ran T, Feig M, de Groot BL, Grubmüller H, MacKerell Jr. AD. 2016. CHARMM36m: An Improved Force Field for Folded and Intrinsically Disordered Proteins. Nat. Methods. 14:71-73.

Jorgensen WL, Chandrasekhar J, Madura JD, Impey RW, Klein ML. 1983. Comparison of Simple Potential Functions for Simulating Liquid Water. J. Chem. Phys. 79:926-935.

Kabsch W, Sander C. 1983. Dictionary of Protein Secondary Structure: Pattern Recognition of Hydrogen-Bonded and Geometrical Features. Biopolymers 22:2577-2637.

Lindorff-Larsen K, Piana S, Palmo K, Maragakis P, Klepeis JL, Dror RO, Shaw DE. 2010. Improved Side-Chain Torsion Potentials for the Amber ff99SB Protein Force Field. Proteins Struct. Func. Bioinformat. 78:1950-1958.

McGibbon RT, Beauchamp KA, Harrigan MP, Klein C, Swails JM, Hernández CX, Schwantes CR, Wang L-P, Lane TJ, Pande VS. 2015. MDTraj: A Modern Open Library for the Analysis of Molecular Dynamics Trajectories. Biophys. J. 109:1528-1532.

Parlitz R, Eitan A, Stjepanovic G, Bahari L, Bange G, Bibi E, Sinning I. 2007. *Escherichia coli* Signal Recognition Particle Receptor FtsY Contains an Essential and Autonomous Membrane-binding Amphipathic Helix. J. Biol. Chem. 282:32176-32184.

Parrinello M, Rahman A. 1980. Crystal Structure and Pair Potentials: A Molecular Dynamics Study. Phys. Rev. Lett. 45:1196-1199.

Ryckaert J-P, Ciccotti G, Berendsen HJC. 1977. Numerical Integration of the Cartesian Equations of Motion of a System with Constraints: Molecular Dynamics of *n*-Alkanes. J. Comput. Phys. 23:327-341.

The Uniprot Consortium. 2018. UniProt: The Universal Protein Knowledgebase. Nucelic Acids Res. 46:2699.

van der Spoel D, Lindahl E, Hess B, Groenhof G, Mark AE, Berendsen HJC. 2005. GROMACS: Fast, Flexible and Free. J. Comp. Chem. 26:1701-1718.
